# Transcranial alternating current stimulation for neuropsychiatric disorders: a systematic review of treatment parameters and outcomes

**DOI:** 10.3389/fpsyt.2024.1419243

**Published:** 2024-08-14

**Authors:** Fatemeh Gholamali Nezhad, Josh Martin, Vanessa K. Tassone, Alyssa Swiderski, Ilya Demchenko, Somieya Khan, Hamzah E. Chaudhry, Annalisa Palmisano, Emiliano Santarnecchi, Venkat Bhat

**Affiliations:** ^1^ Interventional Psychiatry Program, St. Michael’s Hospital - Unity Health Toronto, Toronto, ON, Canada; ^2^ Institute of Medical Science, Temerty Faculty of Medicine, University of Toronto, Toronto, ON, Canada; ^3^ Institute of Biomedical Engineering, Science, and Technology (iBEST), Keenan Research Centre for Biomedical Science, St. Michael’s Hospital - Unity Health Toronto, Toronto, ON, Canada; ^4^ Precision Neuroscience and Neuromodulation Program, Gordon Center for Medical Imaging, Massachusetts General Hospital and Harvard Medical School, Boston, MA, United States; ^5^ Chair of Lifespan Developmental Neuroscience, TUD Dresden University of Technology, Dresden, Germany; ^6^ Neuroscience Research Program, St. Michael’s Hospital - Unity Health Toronto, Toronto, ON, Canada; ^7^ Department of Psychiatry, Temerty Faculty of Medicine, University of Toronto, Toronto, ON, Canada

**Keywords:** transcranial alternating current stimulation, transcranial electrical stimulation, brain stimulation, mental disorders, psychiatry, therapeutics

## Abstract

**Background:**

Transcranial alternating current stimulation (tACS) alters cortical excitability with low-intensity alternating current and thereby modulates aberrant brain oscillations. Despite the recent increase in studies investigating the feasibility and efficacy of tACS in treating neuropsychiatric disorders, its mechanisms, as well as optimal stimulation parameters, are not fully understood.

**Objectives:**

This systematic review aimed to compile human research on tACS for neuropsychiatric disorders to delineate typical treatment parameters for these conditions and evaluate its outcomes.

**Methods:**

A search for published studies and unpublished registered clinical trials was conducted through OVID (MEDLINE, PsycINFO, and Embase), ClinicalTrials.gov, and the International Clinical Trials Registry Platform. Studies utilizing tACS to treat neuropsychiatric disorders in a clinical trial setting were included.

**Results:**

In total, 783 published studies and 373 clinical trials were screened; 53 published studies and 70 clinical trials were included. Published studies demonstrated a low risk of bias, as assessed by the Joanna Briggs Institute Critical Appraisal Tools. Neurocognitive, psychotic, and depressive disorders were the most common disorders treated with tACS. Both published studies (58.5%) and registered clinical trials (52%) most commonly utilized gamma frequency bands and tACS was typically administered at an intensity of 2 mA peak-to-peak, once daily for 20 or fewer sessions. Although the targeted brain locations and tACS montages varied across studies based on the outcome measures and specific pathophysiology of the disorders, the dorsolateral prefrontal cortex (DLPFC) was the most common target in both published studies (30.2%) and registered clinical trials (25.6%). Across studies that published results on tACS outcome measures, tACS resulted in enhanced symptoms and/or improvements in overall psychopathology for neurocognitive (all 11 studies), psychotic (11 out of 14 studies), and depressive (7 out of 8 studies) disorders. Additionally, 17 studies reported alterations in the power spectrum of the electroencephalogram around the entrained frequency band at the targeted locations following tACS.

**Conclusion:**

Behavioral and cognitive symptoms have been positively impacted by tACS. The most consistent changes were reported in cognitive symptoms following gamma-tACS over the DLPFC. However, the paucity of neuroimaging studies for each neuropsychiatric condition highlights the necessity for replication studies employing biomarker- and mechanism-centric approaches.

## 1 Introduction

Transcranial alternating current stimulation (tACS), a variant of transcranial electrical stimulation (tES), involves the delivery of low-intensity alternating current (AC) to the brain to target the excitability of mainly cortical neurons (1). The primary mechanisms of tACS are related to the incorporation of AC-induced oscillations into the endogenous oscillations of the brain, as well as inducing synaptic plasticity (2). Contrary to many types of tES, tACS does not depolarize or hyperpolarize neurons due to the alternating direction of the delivered current (3, 4). The primary mechanism of tACS is related to incorporating AC-induced oscillations into the natural, endogenous oscillations of the brain, as well as inducing synaptic plasticity (2). At the systems level, tACS modulates brain function by affecting connectivity between cortical regions, and cortical excitability (5, 6). Studies investigating tACS have reported changes in brain wave characteristics, connectivity patterns, and associated cognitive processes, demonstrating the correlation between brain oscillations and higher-order functions. Studies investigating tACS have reported changes in brain wave characteristics, connectivity patterns, and associated cognitive processes, demonstrating the correlation between brain oscillations and higher-order functions (2). This is notable as almost all psychiatric disorders have shown abnormalities in electroencephalogram (EEG) oscillatory patterns (7). Based on the ability of tACS to modulate pathological brain rhythms, an increasing number of randomized controlled trials (RCTs) have recently emerged, aiming to test the efficacy of tACS for the treatment of neuropsychiatric disorders (8–10).

Apart from their low cost and relative ease of application, tES modalities provide the opportunity to establish a viable sham condition for double-blind RCTs (11). Such trials have demonstrated the feasibility of tACS in changing brain wave patterns and improving behavioral outcomes without significant adverse events (2). However, a neuroscientific understanding of how to efficiently target brain function and individualize the intervention is lacking (8). Optimization of parameters such as the stimulation frequency, amplitude, target, polarity, and duration (12, 13) is crucial for the success of device-based therapeutic methods, such as tACS.

Although tACS has been used for neuromodulation purposes for many years (14) and the application of tACS in neuropsychiatry seems promising, additional research is still warranted as there are no clear criteria or clinical guidelines for employing tACS as a non-invasive brain stimulation (NIBS) therapy. Probable outcomes and disease trajectories still remain unmapped (13). By synthesizing the existing evidence for tACS, it may become possible to elucidate which treatment parameters lead to better outcomes. Thus, this systematic review aims to address the typical treatment parameters and outcomes of tACS in human subjects with neuropsychiatric disorders. To explore this, the review has set three main goals: (1) to provide an overview and synthesis of current literature on tACS for neuropsychiatric disorders, (2) to identify the most typical and effective parameters for different neuropsychiatric disorders, and (3) to evaluate behavioral and neurobiological treatment outcomes of tACS.

The available systematic reviews on this topic typically focus on published studies (2, 14, 15) and specific tACS paradigms (16), as well as the treatment efficacy of tACS for specific psychiatric disorders (10, 17, 18). However, considering the quickly evolving research landscape, it is worth examining ongoing trials to provide the most up-to-date information on the application of tACS, and eliminate bias created by the overrepresentation of positive results. Moreover, the literature suggests that tACS can improve common symptoms shared by many neuropsychiatric disorders, such as cognitive impairment (19). Thus, it would be beneficial to adopt a broader perspective encompassing all neuropsychiatric disorders and amalgamate the advantages offered by various tACS paradigms. With this work, we aim to provide an up-to-date snapshot of the overall clinical utility of tACS as an intervention for neuropsychiatric disorders.

## 2 Methods

This systematic review followed the Preferred Reporting Items for Systematic Reviews and Meta-Analyses (PRISMA) guidelines (20). The completed PRISMA Checklist is displayed in **Supplementary Table 1**.

### 2.1 Search strategy

First, a preliminary search was conducted in April 2023 to grasp the range of neuropsychiatric conditions for which tACS has been used in a clinical trial setting. Subsequently, a comprehensive systematic search for published studies and registered clinical trials involving the administration of tACS in the identified neuropsychiatric populations was conducted on May 3rd, 2023. The detailed search strategies for the published studies and unpublished clinical trials are provided in **Supplementary Table 2**. Briefly, a search of three databases with published studies, MEDLINE, PsycINFO, and Embase, was conducted through OVID using two search concepts with the Boolean operators “AND” and “OR”. The first search concept was related to the disorder categories and included but was not restricted to *mental disorders* OR psychiatric disorder* OR psychological disorder* OR neurodevelopmental* disorder OR neurocognitive disorder*. The second search concept was related to alternat* current stimulation* OR *tACS* OR alternat* current*. Additionally, a search for registered clinical trials involving tACS was conducted on ClinicalTrials.gov and the International Clinical Trials Registry Platform (ICTRP; https://www.who.int/ictrp/en/) of the World Health Organization. The search term included *alternating current stimulation* OR *tACS.* Search limits/restrictions were not applied. Protocol papers were categorized and reviewed as published studies, and if a registered clinical trial was associated with a publication, the publication was located, and the study was categorized and reviewed as a published study.

### 2.2 Inclusion and exclusion criteria

Two authors (AS and SK or HEC) independently performed first- (i.e., title and abstract) and second-level (i.e., full-text) screening to assess retrieved studies for eligibility. Discrepancies were discussed and resolved by a third party (F.G.) when consensus was not reached. Studies were included if at least one study group received verum tACS. Studies administering tACS in combination with or following other treatments (e.g., tACS + medications; tACS + psychotherapy; tACS + other forms of NIBS) were included. tACS must have been administered for treatment purposes, and non-clinical studies investigating basic brain and cognitive functions were excluded. At least one study group must have represented a disordered population included in the fourth and fifth editions of the Diagnostic and Statistical Manual of Mental Disorders (DSM) (i.e., no studies on solely healthy participants, but including a healthy control group along with patients was acceptable). Participants were also required to be 18 years of age or older. There was no restriction on the year of publication/registration or the sex of participants. Articles were excluded if they were reviews, meta-analyses, or conference publications. The full list of eligibility criteria is presented in **Supplementary Table 3**.

### 2.3 Variable extraction

The extracted variables were classified into eight categories encompassing the study’s identifying features, study status, treated neuropsychiatric condition(s), participant characteristics, study design, study outcomes, treatment parameters, and study results.

#### 2.3.1 Published studies

The year of publication and the country of study were recorded for each published article. Level of evidence (i.e., RCT, case-controlled studies, and cohort studies), allocation (i.e., randomized or non-randomized), intervention model (i.e., single group assignment, parallel assignment, cross-over assignment, and factorial assignment), and masking (i.e., open-label, single-blind, double-blind, triple-blind, or quadruple-blind) were all recorded. Additionally, comparison type, the number of study arms, and administered treatment modalities were noted, including other NIBS (e.g., transcranial direct current stimulation [tDCS], transcranial random noise stimulation [tRNS], transcranial magnetic stimulation [TMS]) or non-NIBS (e.g., physical therapy, cognitive rehabilitation, medication, psychotherapy) treatment types.

The following participant characteristics were also extracted: age group of the sample (i.e., adult or older adult), primary diagnoses, mean age of participants, and percentage of female participants. Information about inclusion age, target sex (i.e., male only, female only, or both), and presence of a healthy control group (i.e., yes or no) were recorded. Total enrollment number and enrollment in verum tACS arm(s) were noted.

Moreover, the following tACS stimulation parameters were recorded: frequency (in Hz), frequency band (i.e., alpha, beta, delta, theta, or gamma), intensity (i.e., peak-to-peak amplitude in mA), stimulation target, single tACS session duration (in minutes), and total number of tACS sessions over the course of treatment. Some studies presented more than one value for each category of treatment parameters (e.g. administering both delta and theta frequency bands to one tACS arm), in which case all provided information was extracted.

The primary and secondary outcomes of each study were noted and categorized into safety and feasibility (e.g., dropout rates, adverse events, participant satisfaction); treatment efficacy (i.e., clinical scales); neuropsychological testing (e.g., cognitive batteries, computerized tasks); radiological brain imaging (e.g., magnetic resonance imaging [MRI]); molecular brain imaging (e.g., positron emission tomography [PET]); and electrophysiological/cortical excitability/magnetic signal recording (e.g., electroencephalography [EEG]). Study outcomes could be listed under more than one. Further, published results pertaining to safety and feasibility, treatment efficacy, radiological or molecular brain imaging, and electrophysiological/cortical excitability/magnetic signal recording were extracted.

#### 2.3.2 Registered clinical trials

The same variables as those detailed above were extracted from the included unpublished clinical trials. In addition, principal investigator, trial registration year, actual or estimated completion year, completion status, and actual or projected intention-to-treat sample sizes were also recorded from the trial entry. If results from a clinical trial had been published, this was noted, and such trials were reviewed as published studies.

### 2.4 Assessment of quality for published literature

Published studies included in the review were assessed for methodological quality (risk of bias) using the Joanna Briggs Institute (JBI) Critical Appraisal Tools for RCTs, Quasi-Experimental Studies (21), Case Reports (22) or Case Series (23). Two authors (AS and SK or HEC) independently performed the quality assessment. Discrepancies were discussed and resolved by a third party (F.G.) when consensus was not reached.

## 3 Results

**Figure 1:**
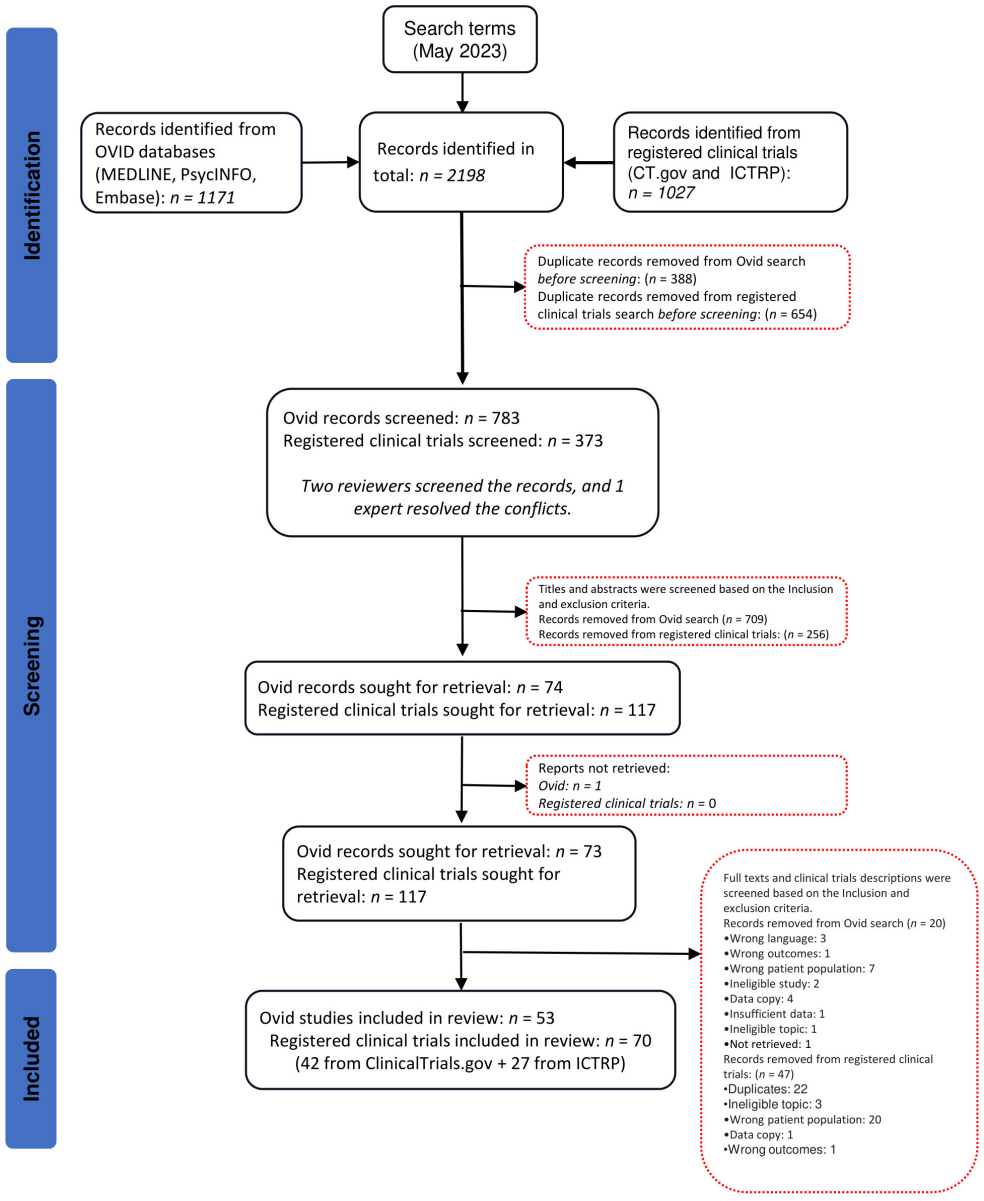
PRISMA diagram.

After conducting the searches and removing duplicate records, 783 published studies and 373 clinical trials were screened at the first level. Of these, 73 published studies and 117 clinical trials were assessed for eligibility at the second level. Published studies and unpublished clinical trials excluded in the second level screening are listed in **Supplementary Table 4**. In total, 53 published studies from OVID and 70 clinical trials (42 from ClinicalTrials.gov + 27 from ICTRP) were included in this systematic review (**Figure 1**).

### 3.1 Quality assessment

The results of the quality assessment for published studies are provided in **Supplementary Table 5**. Among RCTs, 97.2% of studies utilized true randomization for the assignment of participants to treatment groups and had treatment groups that were similar at baseline. For quasi-experimental studies, there were multiple measurements of the outcomes in all studies (100%), with complete follow-up in 75% of studies, and if not, differences between groups in terms of follow-up were adequately described and analyzed. With regards to case report studies, all studies (100%) provided take-over lessons, and they could clearly describe the current and post-treatment clinical condition of the patient, as well as the interventions procedures. Furthermore, among case series, only 37.5% had clear criteria for inclusion. However, for 62.5% of case series, the condition was measured in a standard, reliable way, and valid methods were used for identification of the condition for all the participants.

Among the assessed RCTs, participants, the intervention team, and outcome assessors were blinded to treatment assignment in 88.8%, 72.2%, and 77.7% of studies, respectively. Groups were treated identically, other than the intervention of interest, in all studies (100%). Follow-up was complete, and if not, differences between groups in terms of follow-up were adequately described and analyzed in 61.1% of studies. Participants were analyzed in the groups to which they were randomized in 80.5% of studies. Where applicable, 88.8% of the studies concealed allocation to groups. Appropriate statistical analysis and trial design was used by 94.4% of RCTs.

### 3.2 Studies by publication/registration year and completion status

#### 3.2.1 Published studies

**Figure 2:**
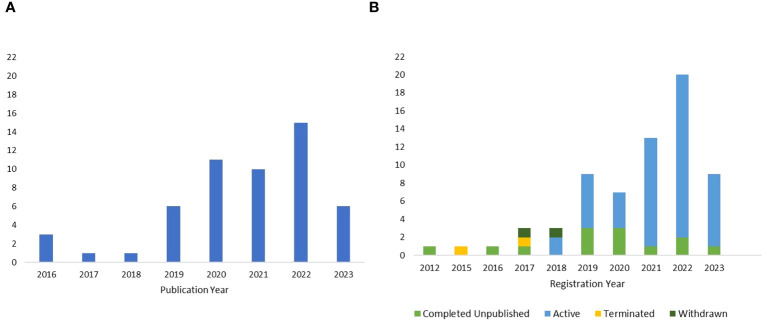
**(A)** Publication year of the completed and published studies until May 3rd, 2023. **(B)** Registration year of the included clinical trials, along with their corresponding completion status.

The published studies were found to span 13 countries across 4 continents (**Supplementary Figure 1A**), with the largest contributor being the United States. The first publications on tACS as treatment for neuropsychiatric disorders emerged in 2016. Since then, the number of published studies applying tACS in neuropsychiatric disorders generally trended upward. The greatest number of completed and published studies (i.e., 15) emerged in 2022 (**Figure 2A**).

#### 3.2.2 Registered clinical trials

For registered clinical trials, research efforts spanned 13 countries situated across 4 continents (**Supplementary Figure 1B**). Notably, the United States was the most prominent contributor. The first tACS trial was registered in 2012, and the number of subsequently registered trials generally trended upward, reaching its peak in 2022. Among the unpublished trials included in the review, 73.6% were active, while 20.6% were marked as completed and unpublished (**Figure 2B**). A smaller percentage of clinical trials, 2.9%, were terminated, and another 2.9% were withdrawn. Only one clinical trial provided reasoning for its termination, noting that the tACS treatment schedule was dense and precluded the successful recruitment of study participants.

### 3.3 Studies by participant characteristics

#### 3.3.1 Published studies

Of 53 published studies, 51 provided an age range for the study populations. A majority of published studies exclusively involved adult participants (56.6%), ranging in age from 18 to 65. Meanwhile, 5.7% of published studies involved older adults aged above 65, and 35.8% of them included both adults and older adults. The mean age of participants was 45.6 (SD = 17.3). Additionally, the majority of the published studies (73.5%) included participants of both genders, with varying ratios across different studies. The other 26.5% exclusively encompassed either females or males or did not report the sex of participants.

#### 3.3.2 Registered clinical trials

Among the 70 trials included, most studies included both older adults and adults (51.4%), followed by only adults (47.1%) then only older adults (1.5%). Only one trial provided details regarding the mean age of enrolled participants. However, the average minimum inclusion age across all registered trials was 30 (SD = 17.4), while the average maximum inclusion age was 69.6 (SD = 13.9). Additionally, 94% of ongoing clinical trials encompassed both males and females, and the remaining 6% were female-only.

### 3.4 Studies by clinical indications

#### 3.4.1 Published studies

**Figure 3:**
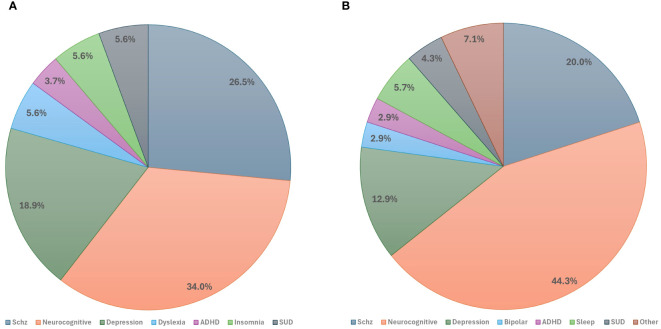
Studies by primary clinical indications: **(A)** Primary indications of published studies. **(B)** Primary indications of registered clinical trials. ADHD, Attention-Deficit Hyperactivity Disorder; Schz, Schizophrenia; SUD, Substance Use Disorder.

The included published studies most commonly (34%) focused on neurocognitive disorders, including Alzheimer’s disease (AD), dementia, mild cognitive impairment (MCI), Parkinson’s disease (PD), and Huntington’s disease (HD). Disorders with psychotic features, including schizophrenia, schizoaffective disorder, and psychotic bipolar disorder, were the second most studied indications (26.5%), followed by disorders with depressive symptoms including MDD, premenstrual dysphoric disorder (PMDD), and internalizing psychopathologies (18.9%) (**Figure 3A**). Other less common indications included substance use disorders (i.e., substance use disorder and tobacco use disorder), neurodevelopmental disorders (i.e., attention deficit hyperactivity disorder and dyslexia), and sleep-wake disorders (i.e., insomnia) (**Table 1**).

**Table 1:** Treatment parameters of published studies.

ID	Primary diagnosis	Description of study arms	tACS frequency (Hz)	Peak-to-Peak amplitude per electrode(mA)	Targeted brain region	Total number of sessions	Duration of each session (min)
Ahn S et al. (2019) (24)	Schizophrenia	tACS/patients, tDCS/patients, sham/patients	10	2	DLPFC and TPJ	10	20
Alexander M.L et al. (2019) (25)	MDD	tACS/patients, tACS/patients, sham/patients	10 vs. 40	2	Left DLPFC	5	40
Assogna M et al. (2021) (26)	Dementia	tACS/patients, sham/patients	40	2	Bilateral frontotemporal	30	60
Benussi A et al. (2021) (27)	AD	tACS/patients, sham/patients	40	3	Precuneus	1	60
Benussi A et al. (2022) (28)	AD	tACS/patients, sham/patients	40	3	Precuneus	1	60
Bréchet L et al. (2021) (29)	AD	tACS/patients	40	2	Left angular gyrus	70	20
Chang C.-C et al. (2021) (30)	Schizophrenia	tACS/patients, sham/patients	6	2	Left frontoparietal	10	20
Dallmer-Zerbe I et al. (2020) (31)	ADHD	tACS/patients, sham/patients	1.5 - 20	1	Parietal and temporal cortices	1	20
Daughters S.B et al. (2022) (32)	SUD	Sham (session 1 and session 2)/patients, sham (session 1) and tACS (session 2)/patients, sham (session 1) and tACS (session 2)/patients	10 vs. 40	2	Bilateral DLPFC	1	40
Davis MC et al. (2023) (33)	HD	tACS, then tACS, then sham/patients	IAF & 2	2	Bilateral mPFC	2	20
Davis MC et al. (2023) (34)	HD	tACS, then tACS, then sham/patients	IAF & 2	2	Bilateral mPFC	2	20
Del Felice A et al. (2019) (35)	PD	tACS + PT, then RNS + PT/patients, tACS + PT, then RNS + PT/patients	30 vs. 4	1-2	Based on the power spectrum	10	30
Dhaynaut M et al. (2022) (36)	AD	tACS/patients	40	2	Bilateral temporal lobes	20	60
Force R.B et al. (2021) (37)	Schizophrenia	tACS/patients	10	1	Left DLPFC	20	40
Haller N et al. (2020) (38)	MDD	tACS/patients	40	2	Bilateral PFC	20 vs. 10	10 vs. 20
Haller N et al. (2020) (39)	Schizophrenia	tACS/patients	40	2	Bilateral DLPFC	10	20
Haller N et al. (2020) (40)	Schizophrenia	tACS/patients	40	2	Bilateral DLPFC	20	10
Hoy K.E et al. (2016) (41)	Schizophrenia	tACS + tDCS + sham/patients	40	2	Left DLPFC	1	20
Huang Y et al. (2021) (42)	MDD	tDCS + placebo/patients, tACS + placebo/patients, escitalopram + placebo/patients, sham + placebo/patients	8 - 12	2	Bilateral DLPFC	10	30
Jacobson N et al. (2022) (43)	MCI, dementia	tACS + cognitive exercises/patients, sham + cognitive exercises/patients	40	1.5	Left DLPFC	20	30
Kallel L et al. (2016) (44)	Schizophrenia	tACS/patients	4.5	2	Bilateral DLPFC	20	20
Kannen K et al. (2022) (45)	ADHD	tACS + sham/patients	1 - 8	1	Parietal and temporal regions	1	20
Kehler L et al. (2020) (46)	Dementia or MCI	tACS + cognitive exercise/patients, cognitive exercise/patients	40	1.5	Bilateral DLPFC	20	30
Kim J et al. (2021) (47)	MCI	tACS + tDCS + sham/patients	40	2	Bilateral DLPFC	1	30
Leite J et al. (2022) (48)	MCI	tACS + cognitive training/patients, sham + cognitive training/patients, tACS + sham cognitive training/patients	IAF	2	Bilateral PFC	15	20
Liu Y et al. (2022) (49)	AD	tACS/patients	40	1.5	Bilateral DLPFC	15	20
Marchesotti S et al. (2020) (50)	Dyslexia	tACS + tACS + sham/patients, tACS + tACS + sham/HC	30, 60	Max of 2	Left auditory cortex	1	20
McAleer J et al. (2023) (51)	Internalizing psychopathologies	tACS/patients, tDCS/patients	6	2	Based on baseline EEG	2	20
Mellin J.M et al. (2018) (52)	Schizophrenia	tACS/patients, tDCS/patients, sham/patients	10	2	Left DLPFC and left TPJ	10	20
Mondino M et al. (2020) (53)	TUD	tACS then sham/patients, sham then tACS/patients	10	2	Bilateral DLPFC	1	30
Motamedi G.K et al. (2023) (54)	Insomnia	tACS + sham/patients	0.75	0.75	Frontal and temporal	10.2 ± 5.7	5
Moussavi Z et al. (2021) (55)	Dementia	Cognitive exercises + tACS/patients, cognitive exercises/patients	40	1.5	Left DLPFC	20	30
Naro A et al. (2016) (56)	MCI, dementia	tACS + sham/MCI, tACS + sham/AD, tACS + sham/HC	40 - 120	1	Left M1, PMA, SMA, DLPFC, and DMPFC	1	6
Palm U et al. (2022) (57)	MDD	tACS/patients, tACS/HC	40	2	Bilateral DLPFC	1	20
Raymond N et al. (2023) (58)	Schizophrenia, schizoaffective or psychotic bipolar disorder	tACS + HD-tDCS/patients, HD-tDCS/patients	2	2	Bilateral eVC	10	20
Riddle J et al. (2020) (59)	MDD	tACS/patients	10	2	Bilateral frontal cortices	17	40
Riddle J et al. (2022) (60)	PMDD	tACS/patients	8 - 12	Unknown	Bilateral frontal cortices	1	40
Riddle J et al. (2022) (61)	MDD	tACS/patients, sham/patients, tACS/HC, sham/HC	8 - 12	2	Bilateral frontal cortices	1	40
Rufener K.S et al. (2019) (62)	Dyslexia	tACS, tRNS, sham/patients	40	1, 1.5	Bilateral auditory cortex	1	0.5
Soleimani G et al. (2022) (63)	SUD	tACS/patients	6	2	Frontoparietal network	1	Unknown
Sprugnoli G et al. (2021) (64)	AD	tACS/patients	40	4	Right temporal lobe	10	60
Sreeraj VS et al. (2020) (65)	Schizophrenia	tACS/patients	10	2	mPFC	10	20
Sreeraj VS et al. (2019) (66)	Schizophrenia	tACS/patients	6	2	Left DLPFC and posterior parietal	5	20
Sreeraj VS et al. (2017) (67)	Schizophrenia	tACS then tACS/patients	6 & 40	2	Left DLPFC and posterior parietal	2	20
Wang H et al. (2022) (68)	MDD	tACS/patients, sham/patients	77.5	15	Frontal and temporal cortices	20	40
Wang H et al. (2020) (69)	Insomnia	tACS/patients, sham/patients	77.5	15	Frontal and temporal cortices	20	40
Werchowski M et al. (2022) (70)	Dyslexia	tACS/patients, tACS/HC	10	1	Left PFC	3	14
Wilkening A et al. (2019) (71)	MDD	tACS/patients	40	2	Bilateral DLPFC	9	20
Xing Y et al. (2020) (72)	AD	tACS/patients, sham/patients	40	15	Frontal and temporal cortices	30	60
Yeh TC et al. (2023) (73)	Schizophrenia	tACS/patients, sham/patients	6	2	Left frontoparietal	10	20
Zhang M et al. (2022) (74)	Schizophrenia	tACS treatment & tACS maintenance/patients, sham tACS treatment & maintenance/patients, sham tACS treatment & tACS maintenance/patients, tACS treatment & sham tACS maintenance/patients	10	2	DLPFC and TPJ	13	40
Zhou D et al. (2022) (75)	Dementia, AD	tACS/patients, sham/patients	40	2	Bilateral temporal lobes	30	20
Zhou Q et al. (2021) (76)	Insomnia	tACS/patients, sham/patients	77.5	15	Frontal and temporal	20	40

#### 3.4.2 Registered clinical trials

The range of conditions for which tACS was utilized was broader in ongoing registered clinical trials compared to published studies. However, consistent with the published studies, the registered trials predominantly addressed neurocognitive disorders including AD, MCI, dementia, PD, and delirium (44.3%). Schizophrenia emerged as the second most researched indication (20%), followed by disorders characterized by depressive symptoms, including MDD, MDD with anxiety, and PMDD (12.9% total) (**Figure 3B**).

Other less commonly studied conditions included sleep-wake disorders (i.e., insomnia and chronic sleep disorder), substance use disorders (i.e., alcohol use disorder, substance dependence, and opioid-use disorder), neurodevelopmental disorders (i.e., ADHD, stuttering, and developmental dyslexia), bipolar disorders (i.e., bipolar disorder), obsessive compulsive disorders (i.e., obsessive compulsive disorder [OCD]) and somatic symptom disorders (i.e., psychosomatic disease) (**Table 2**).

**Table 2:** Treatment parameters of registered clinical trials.

ID	Primary diagnosis	Description of study arms	tACS frequency (Hz)	Peak-to-Peak amplitude per electrode(mA)	Targeted brain region	Total number of sessions	Duration of each session (min)
NCT05496413	Schizophrenia	tACS/patients vs. sham/patients	6	1	DLPFC	1	15
ChiCTR2100042343	Insomnia	tACS/patients vs. sham/patients vs. drug reduction therapy + abdominal acupuncture therapy/patients vs. drug reduction therapy + sham acupuncture/patients vs. no intervention/HC	Unknown	Unknown	Unknown	Unknown	Unknown
NCT05723172	AD	tACS/patients vs. sham/patients	40	Unknown	Unknown	10	40
NCT04088643	AD	tACS/patients vs. sham/patients	4	15	Frontal and temporal cortices	30	60
ChiCTR2200057847	Insomnia or depression and GAD	tACS/insomnia vs. sham/insomnia vs. tACS/anxiety vs. sham/depression anxiety	Unknown	Unknown	Unknown	Unknown	Unknown
ACTRN12619000182190	MCI	tACS/patients vs. sham/patients	40	1	Left PFC	Unknown	20
NCT04770025	AUD	tACS + motivational interviewing/patients vs. sham + motivational interviewing/patients vs. motivational interviewing/patients	15 - 40	Unknown	Unknown	Unknown	30
NCT05445999	Insomnia	tACS/patients	0 - 10	3	DLPFC	10	20
ChiCTR1800016480	Insomnia	tACS/patients vs. sham/patients	77.5	15	Unknown	Unknown	Unknown
DRKS00022927	ADHD	tACS/patients vs. sham/patients	In alpha range	Unknown	Unknown	1	18
NCT05188105	Dementia with Lewy bodies	tACS/patients vs. sham/patients	12	3	Occipital cortex	1	Unknown
NCT03112902	MCI	Standard tACS & nested tACS & sham (in randomized order)/patients vs.standard tACS & nested tACS & sham (in randomized order)/HC	Unknown	Unknown	Unknown	Unknown	Unknown
NCT05643326	AD	tACS/patients vs. sham/patients	40	Unknown	Precuneus	84	Unknown
NCT05693922	MDD	Behavioral activation + tACS/patients vs. behavioral activation + sham tACS/patients	3 - 20	2	Unknown	1	30
NCT05480124	Bipolar disorder	tACS/patients vs. sham/patients	Unknown	PAC	Unknown	Unknown	60
NCT03587012	MCI or AD	Brain fitness app + tACS/patients vs. brain fitness app/patients	Unknown	Unknown	Unknown	40 vs 48	30
NCT05312359	Substance dependence	tACS/amphetamine addiction vs. sham/amphetamine addiction vs. tACS/alcohol addiction vs. sham/alcohol addiction vs. no intervention/HC	5	PAC	DLPFC	20	20
NCT05445466	Bipolar disorder	tDCS/patients vs. sham/patients vs. tACS/patients	Personalized beta-gamma	Unknown	OFC	10	20
ChiCTR2200057944	MDD and anxiety	tACS/patients vs. sham/patients	Unknown	Unknown	Unknown	Unknown	Unknown
NCT05291208	MCI	tACS + cognitive training/patients vs. sham + cognitive training/patients	4-8	2	Prefrontal and parietal regions	8	10
NCT05772702	MDD	tACS/patients	IAF	Unknown	Unknown	5	Unknown
IRCT20230125057214N1	MCI	tACS/patients vs. cognitive rehabilitation/patients vs. tACS + cognitive rehabilitation/patients vs. vitamin D/patients	Unknown	Unknown	Unknown	10	20
NCT04859504	Schizophrenia	tACS/patients vs. sham/patients	7	1-2.4	Right DLPFC and cerebellum	10	20
ChiCTR2000039631	MCI	Electroacupuncture/patients vs. tACS + electroacupuncture/patients vs. sham + electroacupuncture/patients	Unknown	Unknown	Unknown	Unknown	Unknown
NCT04135742	MCI	tACS + cognitive training/patients vs. sham tACS + cognitive training/patients vs. tACS + sham cognitive training/patients	40	2	DLPFC	24	20
ChiCTR2000034547	MDD	tACS/patients vs. sham/patients	In theta range	Unknown	Unknown	Unknown	Unknown
DRKS00011364	Schizophrenia, MDD	tACS/patients vs. sham/patients	40	2	Bilateral frontal cortices	1	20
ChiCTR2000036935	Schizophrenia, schizoaffective disorder, or schizophreniform disorder	tACS/patients vs. tACS/patients vs. sham/patients	Theta vs gamma (unknown exact frequencies)	Unknown	Unknown	Unknown	Unknown
ChiCTR2200062397	MDD	tACS/patients vs. sham/patients	Unknown	Unknown	Unknown	Unknown	Unknown
NCT05282329	Schizophrenia	tACS/patients vs. sham/patients	In gamma range	2	Temporal lobe	20	20
CTRI/2021/11/038189	Schizophrenia	tACS/patients	In theta range	2	Frontoparietal network	10	20
IRCT20210622051675N1	Schizophrenia	tACS + tACS + sham/patients vs. tDCS + tDCS + sham/patients	8 & 40	Unknown	Left frontal cortex	3	20
IRCT20181215041975N1	Stuttering	tDCS + speech therapy/patients vs. tACS + speech therapy/patients	Unknown	Unknown	Based on baseline EEG	10	30
NCT03062553	Schizophrenia	Metacognitive training + tACS/patients vs. sham + metacognitive training/patients vs. tACS/patients	Varied based on baseline EEG	Unknown	Based on baseline EEG	Unknown	Unknown
NCT04647032	MCI	tACS + cognitive control training/patients vs. tACS/patients	6 vs. 1	Unknown	PFC	8	Unknown
NCT04646499	MCI	tACS/patients	40	Unknown	Unknown	8	60
NCT03907644	OUD	tACS/patients vs. sham/patients	6	2	Middle frontal gyrus	1	20
NCT03880240	AD	2 weeks of daily tACS/patients vs. 4 weeks of daily tACS/patients vs. 4 weeks of twice daily tACS/patients vs. 2/4 weeks of sham tACS/patients	40	Unknown	Based on PET imaging	10, 20, 40	60
NCT04783350	AD	tACS/patients	40	Unknown	Left angular gyrus	20 or 70 (based on cognitive improvements after 4 weeks)	20
ACTRN12620000558921	Anxiety	tACS + sham/patients vs tACS + sham/HC	Unknown	Unknown	Bilateral DLPFC	2	10
NCT05708001	MCI	tACS then sham/patients	40	Max 2	Memory network	Unknown	Unknown
NCT05084924	MDD	tACS/patients vs. tACS/patients vs. sham/patients	3 - 20 vs. 5 - 50	2	PFC	Unknown	Unknown
ACTRN12619000010190	AD	tACS/patients vs. sham/patients	40	0.057	Left DLPFC	20	20
NCT05808504	PD	tACS or sham/patients vs. tACS or sham/HC	Unknown	2	Frontal sites	Unknown	12-15
ChiCTR2200063729	Chronic insomnia	tACS/patients vs. sham/patients	Unknown	Unknown	Unknown	Unknown	Unknown
DRKS00025122	MCI	tACS + cognitive training/patients vs. sham + cognitive training/patients vs. tACS + cognitive training/HC vs. sham + cognitive training/HC	6 & 80	2	DLPFC	16	20
NCT03994081	MDD	tACS/patients vs. sham/patients	10	2	Unknown	Unknown	40
NCT04785053	AD	tACS, then tACS, then sham/patients vs. tACS, then tACS, then sham/HC adults vs. tACS, then tACS, then sham/HC older adults	Theta, gamma (unknown exact frequencies)	Unknown	Left angular gyrus	3	20
NCT05680701	MCI	tACS, sham tACS/patients vs. tACS + sham tACS/HC	10	4	Unknown	1	20
CTRI/2022/05/042868	Schizophrenia	tACS/patients vs. sham/patients	10	Unknown	Unknown	10	Unknown
NCT03518996	Delirium	TMS/tACS/patients vs. TMS/tACS/HC vs. sham TMS/tACS/patients vs. TMS/tACS/HC	Theta	Unknown	Cerebellum	15	Unknown
ACTRN12612000217808	Schizophrenia	tDCS/patients vs. tACS/patients vs. sham/patients	30 - 40	1	Left DLPFC	1	20
NCT05661084	Dementia or MCI	tACS + tDCS/patients vs. tACS + sham tDCS/patients vs. sham tACS + tDCS/patients vs. sham tACS + sham tDCS/patients	Unknown	Unknown	Left angular gyrus	20	30
NCT05326750	AD	tACS/patients vs. sham/patients	40	3	Precuneus	4	Unknown
NCT04986787	MCI	tACS - within-frequency/HC vs. tACS - cross-frequency/HC vs. cerebellar rTMS + tACS/HC vs. TIS + rTMS/patients	In theta range	Unknown	Frontoparietal network	Unknown	Unknown
NCT05583136	Developmental dyslexia	tACS + visuo-attentional training/patients vs. sham + visuo-attentional training/patients vs. sham + phonics training/patients	Unknown	Unknown	Magnocellular-dorsal visual stream	Unknown	Unknown
NCT04856657	Schizophrenia	tACS at IAF, IAF + 2 Hz, and IAF - 2 Hz/patients vs. tACS at IAF, IAF + 2 Hz, and IAF - 2 Hz/HC	IAF + 2 vs. IAF - 2 vs IAF	1-2	Back of the head (Oz electrode)	Unknown	10-20
NCT02362191	PMDD	tACS/patients	10	Left prefrontal cortex	Unknown	2	40
NCT03351452	MCI, AD	tDCS vs. tACS vs. sham/patients vs tDCS vs. tACS vs. sham/HC	Unknown	2	VLPFC	1	20
ChiCTR2100041850	AD	tACS/patients vs. sham/patients	Unknown	Unknown	Unknown	Unknown	Unknown
ACTRN12620000748910	OCD	tACS/patients vs. sham/patients	10	1.5	mPFC	39	Unknown
DRKS00010907	ADHD	tACS/patients vs. sham/patients	Unknown	1	Unknown	Unknown	20
NCT03756610	Schizophrenia	tACS + active boosting/patients vs. tACS + sham boosting/patients vs. sham + sham boosting/patients	40	2	Left DLPFC	10	20
ChiCTR1800018370	Dementia	tACS/patients vs. sham/patients	Unknown	Unknown	Unknown	Unknown	Unknown
NCT05544201	AD with sleep disturbance	HD-tACS/patients vs. HD-tACS/patients vs. sham/patients	40	2	Left DLPFC	Unknown	20
ChiCTR2200064124	MDD	tACS/patients vs. sham/patients	Unknown	Unknown	Unknown	Unknown	Unknown
NCT05342727	Schizophrenia	tACS, tACS, sham/patients vs. tDCS, tDCS, sham/patients	8 & 40	Unknown	Left DLPFC	2	20
NCT05678725	PD	tACS/patients vs. tDCS/patients vs. sham/patients	20	2	Primary motor cortex	1	20
ACTRN12621001649808	Schizophrenia	tACS/patients vs. sham/patients	5	1.75	Right TPJ	1	16
NCT05710549	MCI	tACS 1 + tACS 2 + sham/patients	Gamma vs. beta (unknown exact frequencies)	Unknown	Autobiographical memory network	3	20

ABM, Attentional bias modification paradigm; AD, Alzheimer’s disease; ADHD, Attention-deficit/hyperactivity disorder; AUD, Alcohol use disorder; DLPFC, Dorsolateral prefrontal cortex; DMPFC, Dorsomedial prefrontal cortex; EEG, Electroencephalogram; eVC, Extrastriate visual cortex; HC, Healthy controls; HD, High-definition; GAD, Generalized anxiety disorder; IAF, Individual alpha frequency; M1, Primary motor cortex; MCI, Mild cognitive impairment; MDD, Major depressive disorder; mPFC, medial prefrontal cortex; OCD, Obsessive compulsive disorder; OFC, Orbitofrontal cortex; OUD, Opioid use disorder; PD, Parkinson’s disease; PMDD, Premenstrual dysphoric disorder; PFC, Prefrontal cortex; PAC, Peak phase-amplitude coupling; PET, Positron emission tomography; PT, Physical therapy; PMA, Premotor area; rTMS, Repetitive transcranial magnetic stimulation; SMA, Supplementary motor area; SUD, Substance use disorder; tACS, Transcranial alternating current stimulation; tDCS, Transcranial direct current stimulation; TMS, Repetitive transcranial magnetic stimulation; TPJ, Temporoparietal junction; tRNS, Transcranial random noise stimulation; TUD, Tobacco use disorder; VLPFC, Ventrolateral prefrontal cortex.

### 3.5 Studies by design and enrollment

#### 3.5.1 Published studies

**Figure 4:**
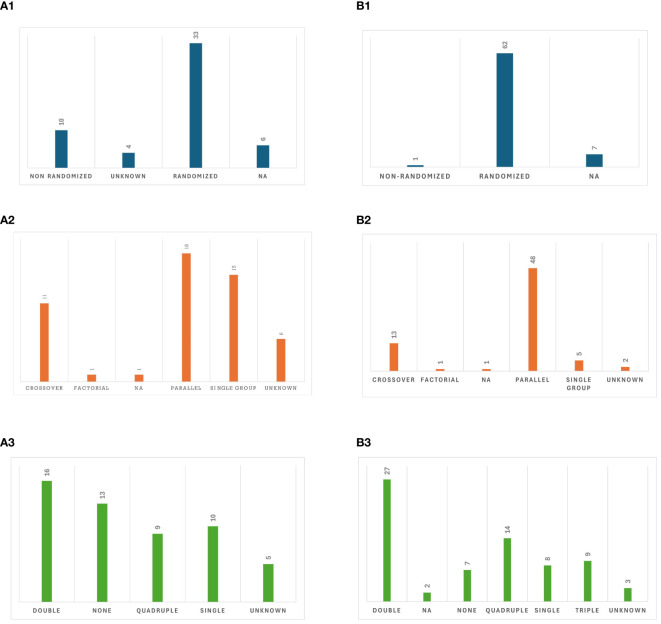
Study design: **(AI)**. Allocation in published studies, **(A2)**. Intervention model in published studies, **(A3)**. Masking in published studies, **(B1)**. Allocation in registered clinical trials, **(B2)**. Intervention model in registered clinical trials, **(B3)**. Masking in registered clinical trials.

Most published studies were interventional RCTs (60.4%) and among different intervention models, parallel assignment (34%) was the most commonly utilized. The details on the design of published studies are presented in **Figure 4A**. Almost a quarter (22.6%) of published studies reported the use of other treatment techniques (NIBS or non-NIBS) as part of the study arms. Among 7 studies that included tDCS in their study arms, 3 compared tDCS to tACS, 3 had tDCS and tACS in the same study arm to augment the stimulation effects. Additionally, 2 studies administered tACS and tRNS in combination with physical therapy or sham stimulation in different orders, and 4 studies compared the effects of cognitive rehabilitation with tACS to sham stimulation with cognitive rehabilitation or cognitive rehabilitation alone. The average number of participants enrolled was 27.5 (SD = 25.2), with a mean enrollment of 18.7 (SD = 17.9) in the tACS group. On average, 96.6% (SD = 6.2%) of enrolled participants completed the entire trial.

It is noteworthy that of 53 published studies, 30 studies included sham arms with either parallel or crossover assignments. Two studies combined sham tACS with cognitive exercise using processing speed training (48) and an attentional bias modification paradigm (53). All studies with sham arms employed single, double, triple, or quadruple masking, except for three (29, 33, 62) that had unblinded or unknown masking status. For the other 23 published studies, tACS was either administered to a single group, compared between two tACS groups, compared with other treatments, or compared with a control group (no tACS for healthy participants or those with similar disorders).

#### 3.5.2 Registered clinical trials

Out of the 70 registered clinical trials, most (88.57%) were randomized, while seven (10.00%) did not specify whether they were randomized or nonrandomized. There was a diverse range of intervention models utilized, among which parallel assignment (68.6%) was the most common. Details on study designs of ongoing clinical trials are provided in **Figure 4B**. Out of 56 trials that provided enrollment information, the average estimated enrollment across these trials (n = 4523) was 80.7 (SD = 69.8). Among the 21 trials that provided actual enrollment data, the mean actual enrollment across these trials (n = 711) was 33.9 (SD = 36.5).

Of out of the 70 clinical trials, 22 used other treatment techniques in their study arms in combination with tACS or in a separate arm. Among them, 10 studies reported use of other NIBS treatments: either tDCS (n = 8), repetitive transcranial magnetic stimulation (rTMS) and temporal interference (n = 1), or rTMS alone (n = 1). Different cognitive rehabilitation techniques, as well as behavioral activation, speech therapy, abdominal acupuncture therapy, and electroacupuncture were included in study arms to augment the therapeutic effects of tACS or to be compared with tACS.

### 3.6 Studies by treatment parameters

#### 3.6.1 Published studies

**Figure 5:**
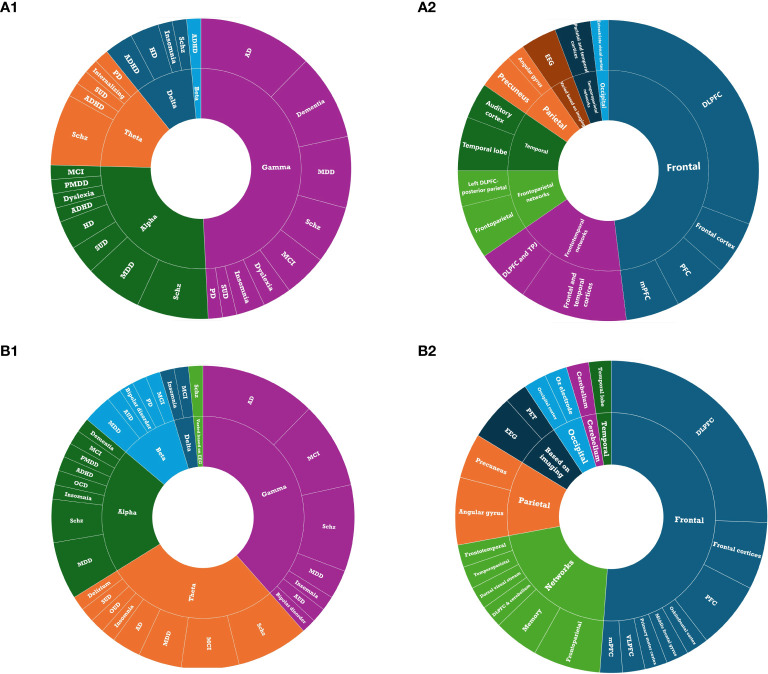
Treatment parameters: **(A1)** Stimulation frequency of tACS in published studies. **(A2)** Stimulation target of tACS in published studies. **(B1)** Stimulation frequency of tACS in registered clinical trials. **(B2)** target of tACS in registered clinical trials.

Across all published tACS studies, the gamma frequency band was the most frequently utilized stimulation frequency band (52.0%) alone or in combination with other frequency bands, followed by alpha (32.1%; **Figure 5A1**). The dorsolateral prefrontal cortex (DLPFC) was the most commonly targeted area (30.2%) for all indications (**Figure 5A2**). Regarding the number of visits, across all the published studies, tACS was mostly administered for either one session, 10 or 20 sessions, once daily and often conducted five days a week. Moreover, except for two studies on insomnia (69, 76), one study on AD (72), and one study on MDD (68), other studies administered a peak-to-peak amplitude of less than 4 mA.

##### 3.6.1.1 Neurocognitive disorders

For studies on neurocognitive disorders, 83.3% used the gamma frequency band with the peak-to-peak amplitude mostly varying from 1 to 3 mA. Interestingly, 40 Hz gamma was the only stimulation frequency applied for AD, dementia, and MCI (at 1.5-2 mA), except for one study (48) which had individualized alpha frequency (IAF) as the stimulation frequency for MCI. The tACS target locations for AD were not consistent across studies (i.e., superior parietal cortex, left angular gyrus, bilateral temporal lobes, DLPFC, right temporal lobe, and frontal and temporal cortices). However, for dementia and MCI, the DLPFC was the most common target area of the brain. Two studies on HD both applied IAF & delta (2 Hz) stimulation (concurrent with physical activity) at a 2-mA intensity, targeting bilateral medial prefrontal cortices (mPFC). Additionally, the only published study on PD compared the effects of gamma tACS to theta tACS for this indication.

##### 3.6.1.2 Disorders with psychotic features

Regarding studies on disorders with psychotic features, the most commonly applied frequencies were theta and alpha, followed by gamma, administered at 1-2 mA peak-to-peak amplitude. The most commonly targeted area was the DLPFC (71.4%) followed by the temporoparietal junction (TPJ).

##### 3.6.1.3 Disorders with depressive symptoms

Moreover, the main target areas for depressive symptoms were located in the frontal lobes (i.e., DLPFC, PFC, frontal cortices), to which gamma and alpha frequency bands with a mostly peak-to-peak amplitude of 2 mA were delivered. There was one study (68) which delivered 77.5 Hz gamma to the both frontal and temporal cortices with a 15-mA peak-to-peak amplitude to assess response and remission rates in drug-naive patients with MDD. For other conditions, the treatment parameters are provided in **Table 1**.

#### 3.6.2 Registered clinical trials

Consistent with the published studies, gamma was the most commonly administered frequency band (58.5%; **Figure 5B1**) and the DLPFC was the most frequent target location (25.6%; **Figure 5B2**) among all clinical trials that reported target location. Ongoing clinical trials appear to be more interested in imaging techniques to define the target locations for tACS (**Table 2**). Regarding the frequency of stimulation sessions, there were fewer single-session tACS protocols relative to those reported in the literature, with more studies opting for tACS protocols that lasted for over 20 sessions.

##### 3.6.2.1 Neurocognitive disorders

In clinical trials involving neurocognitive disorders, the gamma frequency band and a 2-mA amplitude for stimulation were commonly used; however, for studies on AD, peak-to-peak amplitudes higher than 2 mA and up to 15 mA were also seen. For this indication, many trials did not mention their target locations (i.e., unknown) or did not specify the exact area(s) (e.g. occipital lobe in general). In studies on MCI, AD, and dementia, PFC, DLPFC, precuneus, and left angular gyrus, as well as memory networks, frontotemporal networks, and frontoparietal networks were targeted. Also, one study on dementia with Lewy bodies (NCT05188105) targeted the occipital lobe with tACS to improve cognitive function in this population following enhancement in alpha oscillations.

##### 3.6.2.2 Disorders with psychotic features

In clinical trials that investigated disorders with psychotic features, the most commonly used frequency bands were gamma, alpha, and theta, respectively, which were utilized with peak-to-peak amplitudes typically set between 1 to 2 mA. The primary focus of stimulation in these cases was the frontal areas (i.e., DLPFC, bilateral frontal cortices) of the brain.

##### 3.6.2.3 Disorders with depressive symptoms

For trials involving disorders characterized by depressive symptoms, alpha was the predominant frequency band and frontal regions were predominant stimulation targets among studies that specified the stimulation frequency and target locations. The peak-to-peak amplitude for this indication varied from 1 to 15 mA. In the case of other conditions, the treatment parameters varied considerably or there was a lack of comprehensive data on the specific parameters of electrical stimulation used in the trial.

### 3.7 Studies by outcome measures and published results

#### 3.7.1 Published studies

Of the 53 published studies, 5 were protocol papers and 48 reported results on study outcomes. Out of 29 studies that reported the results on adherence to treatment, 8 studies reported 0 dropouts and/or withdrawals from the tACS arm, and 1 study reported mandatory social distancing during the pandemic as the reason for dropouts from the tACS arm. Moreover, no significant serious adverse events were reported in active tACS groups, and this intervention was mostly well-tolerated. All reported adverse events were common and mild-to-moderate. Scalp irritation and pain, discomfort, burning sensation, and redness were limited to the electrode sites. Flashes of light, visual changes, headache, neck pain, dizziness, nausea, phosphene perception, flickering and pricking sensation, sleepiness, trouble concentrating, aurium tinnitus, tinnitus cerebri, anxiety, and fatigue were also reported temporarily during the tACS visit or shortly after the visit.

##### 3.7.1.1 Neurocognitive disorders

Regarding neurocognitive disorders, all 13 studies that reported on the efficacy of tACS consistently revealed improvements in the primary or secondary outcomes, which were primarily measured using clinical and cognitive assessment scales. One particular study (46) applied gamma-tACS combined with brain exercise at the intensity of 1.5 mA on the DLPFC for older adults with dementia, with a schedule of twice-daily 30-minute sessions for 4 weeks. Cognitive enhancement was shown following both gamma-tACS combined with challenging brain exercises and challenging brain exercises only, but there was superior maintenance in the tACS group at the 1-month follow-up visit. Moreover, Moussavi et al. (55), with the same stimulation frequency, amplitude, and schedule of treatment for dementia, reported significant cognitive improvements following gamma-tACS combined with cognitive exercise, although these benefits were more evident at the 1-month follow-up visit than immediately after treatment. Conversely, Zhou et al. (75) showed cognitive improvements in the gamma-tACS group of patients with dementia and AD, measured by Mini-Mental State Examination and Alzheimer’s Disease Cognitive Component Assessment (ADAS-Cog) scores, but noted that ADAS-Cog scores reverted to baseline levels 4 months after gamma-tACS treatment. They applied 30 sessions of daily 40 Hz gamma-tACS for 20 minutes over the bilateral temporal lobes at the intensity of 2 mA. The impacts of gamma-tACS on spectral EEG for neurocognitive disorders were also reported in several other studies. Gamma-tACS of the right temporal lobe for 10 60-minutes sessions at the intensity of 4 mA affected spectral power around the stimulation frequency compared to the rest of the spectrum (64) in patients with AD. Additionally, gamma-tACS of the bilateral temporal lobe for 20 60-minute sessions at the intensity of 2 mA revealed a consistent increase in gamma spectral power throughout each week of tACS stimulation, accompanied by a partial reset during weekends when stimulation was paused, but no changes in overall cognitive function was observed (36). Moreover, a significant correlation between gamma-tACS power magnitude and cognitive improvements was reported in patients with MCI and AD, and healthy controls (56). Additionally, a single session of gamma-tACS for 60 minutes over the precuneus for AD led to significant improvements in memory tasks. Specially, increases in gamma frequencies in the parietal lobes were strongly correlated with cognitive improvement; however, the same correlation was not observed with the frontal, temporal or occipital regions (28). Additionally, compared to tDCS and sham groups, an increased activation of frontal electrodes in the beta band was observed following a single session of gamma-tACS over the DLPFC at peak-to-peak amplitude of 2 mA in MCI (47). Notably, a reduction of beta rhythm following theta-tACS over different brain regions at different time points (i.e., right sensorimotor and left parietal area after treatment and over right sensorimotor and left frontal area at follow-up visit) was shown for PD in a study with individualized stimulation frequencies (35). In this study for participants with beta excess on EEG, stimulation frequency was set at 4 Hz, and for participants with theta excess, it was set at 30 Hz. It was concluded that individualized tACS frequency improves motor and cognitive performance in PD. Along with changes in EEG, reports demonstrated that short-latency afferent inhibition (SAI), an indirect measure of cholinergic transmission evaluated with TMS, had a significant effect of treatment (28). Moreover, PET scans revealed supra-threshold decreases of intracerebral p-Tau burden in regions of the temporal lobe targeted by tACS (36) and a decrease in the ratio of Aβ following tACS, compared to baseline (75). Additionally, gamma-tACS of bilateral temporal lobes was associated with a significant increase in blood perfusion of that area in patients with AD. Moreover, the changes in blood perfusion were positively correlated with changes in spectral power of the gamma band as well as changes in episodic memory (64). Gamma-tACS of right temporal lobe at the intensity of 4 mA for 10 60-minute sessions also led to a significant increase in blood perfusion in the bilateral temporal lobes for AD. Moreover, changes of perfusion were in a positive correlation with changes in cognitive performance as well as changes in spectral power of the gamma frequency band (64). It is important to highlight a study that provided preliminary evidence to support the feasibility and safety of a home-based tACS protocol, consisting 70 sessions of gamma-tACS over the left angular gyrus for older adults with AD (29). This protocol could potentially prevent memory decline in patients. The study proposed that the left angular gyrus, a component of the brain’s memory network, might be an effective target for modulating the aberrant brain oscillations associated with memory difficulties in older adults.

It is also worth mentioning that two studies applied two sessions of tACS over the bilateral mPFC for HD. Participants went through one session of IAF-tACS and one session of 2 Hz delta-tACS, each for 20 minutes at the peak-to-peak amplitude of 2 mA, with a randomized order. One of these two studies which aimed to assess the efficacy of tACS for apathy in HD reported increased amplitude of contingent negative variation amplitude in response to alpha-tACS in HD, but not following delta-tACS (33). The other study found no changes in oscillatory power and functional connectivity following tACS conditions but alpha-tACS increased delta power in neurotypical controls, and delta-tACS increased theta power and theta functional connectivity in neurotypical controls (34). Authors from one of the published protocol papers will conduct a multi-site, randomized, double-blind, placebo-controlled trial on gamma-tACS for patients with dementia, with thirty 60-minute sessions and a 6-month follow-up. Apart from safety and feasibility as primary outcomes, data on different neuropsychological and behavioral assessments, PET scans, MRI, resting state EEG, ERPs, and blood biomarkers will be collected.

##### 3.7.1.2 Disorders with psychotic features

Eleven out of 14 studies on psychotic disorders reported positive changes in their primary or secondary outcomes following tACS. These published studies utilized clinical scales to assess positive and/or negative symptoms of schizophrenia, as well as other outcomes such as quality of life, sleep disturbance, and anxiety levels. Zhang et al. (74) found enhancement in alpha oscillations and reductions in the general psychopathology of schizophrenia with auditory hallucinations following thirteen 40-minute sessions of alpha-tACS over the DLPFC and TPJ, while the auditory hallucinations did not improve. In contrast, Ahn et al. (24) demonstrated that the same tACS parameters delivered over 10 twice-daily sessions improved Auditory Hallucination Rating Scale (AHRS) scores while also enhancing alpha oscillations and functional connectivity and 40 Hz auditory steady-state response. However, in a case-controlled study, Force et al. (37) reported no significant changes in AHRS total scores following 20 weekly 40-minute alpha-tACS sessions over the DLPFC, however, there were positive changes in duration and controllability of auditory hallucinations were shown. Additionally, Sreeraj et al. (65) demonstrated a significant reduction in severity of delusions following ten 20-minute sessions of alpha-tACS over the mPFC at 2 mA peak-to-peak amplitude. Mellin et al. (52) reported that the changes into the AHRS and Positive and Negative Syndrome Scale (PANSS) outcomes were not significantly different between the three arms (alpha-tACS vs. tDCS vs. sham); however, the largest effect size was in the alpha-tACS group.

Utilizing gamma-tACS over the DLPFC at a 2 mA intensity, two case-controlled studies (39, 40) demonstrated improved PANSS and Brief Assessment of Cognition in Schizophrenia scores as well as negative symptoms in patients with schizophrenia; however, they differed regarding the total number of sessions and the duration of each tACS visit. Consistent with these findings, a case series by Kallel et al. (44) showed improvements in negative symptoms and PANSS scores following twenty 20-minute sessions of theta-tACS in clozapine-resistant schizophrenia with negative symptoms. Additionally, it was demonstrated that functional connectivity of theta frequency band, related to negative symptoms of schizophrenia, could be modulated by in-phase theta-tACS (73). In a case report (66), five 20-min sessions of theta-tACS over the DLPFC resulted in an improvement in different cognitive skills including working memory, attention, processing speed, and emotional processing, which were sustained in the follow-up assessment after 50 days. A single session of theta-tACS following a single session of gamma-tACS led to the same results for cognitive improvement in another case report (67). It should be noted that in a study (58) the efficacy of high-definition (HD)-tACS was compared to HD-tDCS for general psychopathology of schizophrenia. Ten sessions of delta-tACS over bilateral extrastriate visual cortex (eVC) for 20 minutes at the intensity of 2 mA resulted in better long-term changes, while HD-tDCS caused short-term positive changes. Nevertheless, the neuroplastic changes of event-related potentials were only observed in the tDCS group. It is worth mentioning that 2 studies (41, 52) on schizophrenia did not report any changes to the positive and negative symptoms of schizophrenia. One of them found no significant differences in behavioral outcomes between alpha-tACS, tDCS, and sham groups (52). Ten Hz alpha-tACS and tDCS were administered peak-to-peak of 2 mA for 5 consecutive days, and AHRS and PANSS were used as outcomes measures. Also, Hoy et al. (41) showed that cognitive improvement was significantly greater following tDCS compared to gamma-tACS, and there was no significant difference between sham and gamma-tACS.

##### 3.7.1.3 Disorders with depressive symptoms

Regarding studies investigating the feasibility and efficacy of tACS for conditions characterized by depressive symptoms, 7 out of 8 studies that published results on study outcomes observed improvements in depressive symptoms following tACS. In a case series of six patients with MDD (38), 40 Hz gamma-tACS was administered in two orders over ten days: two 10-minute stimulations or one 20-minute stimulation per day. In both study groups, depression scores and cognitive function improved. Additionally, drug-naive patients with MDD who received 20 daily 40-minutes sessions of 77.5 Hz gamma-tACS at peak-to-peak amplitude of 15 mA on frontal and temporal cortices had significantly higher response and remission rates, as well as decrease in depression scores, compared to the sham group (68). Interestingly, gamma-tACS for MDD during pregnancy also led to improvement in moderate-to-severe depressive symptoms during treatment and at follow-up, as well as better performance on cognitive tasks (71). In a study (25) that compared 40 Hz gamma-tACS with 10 Hz alpha-tACS, clinical symptom improvements and changes in alpha oscillations were assessed in MDD patients randomly assigned to receive five daily 40-minute sessions of either alpha-tACS, gamma-tACS, or active sham stimulation over the left DLPFC at the intensity of 2 mA. The results revealed a higher number of responders and a significant reduction in alpha power over the left frontal brain regions, as measured by EEG, following alpha-tACS in comparison to gamma-tACS and active sham stimulation.

A case report (59) that applied seventeen 40-minute sessions of alpha-tACS for a patient with a lifelong history of depression reported that the patient responded to treatment after 8 weeks based on the clinical assessments. The patient was in remission after twelve weeks. In another study (60) which applied a single session of alpha-tACS over the bilateral frontal cortices for 40 minutes, it was found that the decreased amplitude of prefrontal midline alpha during the late luteal phase in patients with PMDD was modulated following bilateral alpha-tACS. This study demonstrated that in patients with PMDD, left frontal IAF power decreased following active tACS compared to the sham. However, further analysis revealed that without active tACS, the left frontal IAF power was increased, but this rise in power was negated by IAF-tACS. In another study (61) on target engagement of bifrontal IAF-tACS, it was demonstrated that a single session of IAF-tACS significantly reduced the power of left frontal alpha during the resting state compared to sham in patients with MDD. Notably, one study conducted by Palm et al. (57) administered a single session of gamma-tACS and sham treatment to MDD patients and healthy volunteers, but failed to demonstrate improvements in reaction time and working memory accuracy in both groups. There was also one protocol paper (42) aiming to compare alpha-tACS with tDCS and escitalopram for patients with MDD. This study will apply alpha-tACS over the bilateral DLPFC at 2 mA intensity for 30 minutes over 10 sessions to compare the response rate of these three techniques.

It is worth mentioning that one study on ADHD (45) and three studies on dyslexia (50, 62, 70) reported results on changes of resting-state or task-based EEG after tACS. Kannen et al. (45) administered one day of alpha-tACS at 1 mA intensity over parietal and temporal regions and one day, and a sham on another day. Using a visual oddball task, they demonstrated that while the amplitude of N700 component of ERP increased after active tACS compared to sham, the amplitude of P300, the power of low frequency bands, or neuropsychological performance did not change. Additionally, applying 30 Hz gamma-tACS over the auditory cortex of adults with dyslexia resulted in significantly improved phonological processing and reading accuracy as well as decreased gamma activity in the right superior temporal cortex (50). Moreover, 40 Hz gamma-tACS of auditory cortex improved phoneme categorization and amplitude of P50-N1 complex in adults with dyslexia compared to sham (62). However, IAF-tACS over the left prefrontal cortex resulted in no significant changes in phonological task and did not demonstrated significant modulatory effects of IAF-tACS for this indication (70).

#### 3.7.2 Registered clinical trials

A portion of the clinical trials (27%) was primarily focused on safety aspects of tACS. Importantly, all of these trials reported also assessing other outcomes, including clinical, neuropsychological, and neurobiological outcomes. More than half of clinical trials (60%) included EEG metrics (i.e., resting state EEG, task-based EEG, electrical field EEG, TMS-EEG, or polysomnography) as either their primary or secondary outcomes. Investigating EEG changes was not confined to specific conditions; however, trials targeting AD, MCI, and schizophrenia tended to monitor post-tACS EEG changes. In the realm of neurocognitive disorders, there was an increased interest in collecting radiological imaging data (i.e., structural and functional MRI) along with EEG. Among 16 studies that had neuroimaging as their primary or secondary outcome, 10 were investigating treatment outcomes of tACS for neurocognitive disorders. The remaining 6 focused on participants with MDD, schizophrenia, psychosomatic disease, and opioid-use disorder. There were two clinical trials on AD aiming to concurrently collect heart rate variability along with EEG metrics and neuropsychiatric measures (NCT05723172) or PET amyloid burden and PET tau deposition, along with EEG metrics (NCT03880240).

## 4 Discussion

This systematic review aimed to review the current literature on tACS for neuropsychiatric disorders, evaluate treatment outcomes of tACS, and identify the most typical tACS parameters for these conditions. A total of 53 published studies and 70 clinical trials were reviewed. Studies largely varied with respect to primary diagnosis, treatment protocol parameters (including the stimulation frequency, the montage, the target location, number of visits, and duration of each visit), adjacent treatments administered in the tACS arms, treatment outcome metrics, design, comparison type, and participant characteristics. A meta-analysis was not considered feasible as guidelines suggest that meta-analyses should only be conducted when studies are homogeneous in terms of participants, design, and outcomes (77).

The number of published and newly registered studies in this realm has continued to grow rapidly, especially since 2018. This popularity is likely due to the adaptability, tolerability, and cost-effectiveness of tACS compared to other NIBS techniques (78). It has also been revealed that various cognitive and sensory deficits in psychiatric disorders are associated with abnormal brain oscillations; thus, the ability of tACS to modulate brain activities may provide insight into the causal links between neuronal oscillations and cognitive processes in psychiatric disorders (79). Additionally, applying tACS at investigated amplitudes is generally perceptible to participants, with varying thresholds, which allows for the inclusion of an active sham arm, in a double-blind RCT. The increased number of ongoing sham-controlled RCTs on tACS and neuropsychiatric disorders underscores the growing optimism for the use of tACS in treating these conditions, as well as the importance of determining the optimal tACS parameters. Out of 53 published papers, 30 studies included active sham arms with randomized allocation, mostly maintaining blinding and identical treatment for all groups. However, eight RCTs failed to keep the assessor blind or did not report on it. Studies without a sham group should be interpreted with caution, as they may overestimate efficacy due to placebo effects and lack of blinding, potentially confounding results (15). Additionally, it is noteworthy to mention that although some published studies and ongoing registered clinical trials directly compared different stimulation frequencies, most RCTs were sham-controlled without an active control condition. Thus, to compare the efficacy of stimulation frequencies, an indirect comparison was used in this systematic review. Indirect comparisons are valuable when direct trials are unavailable, allowing for cost-effective evidence synthesis from various studies. However, they can be limited by potential biases due to heterogeneity in study design, populations, and outcomes (80).

Neurocognitive disorders were the most commonly targeted conditions for tACS in both published and newly registered studies. The significant prevalence of neurocognitive disorders highlights the pressing requirement for intervention techniques, which are ideally portable, adaptive and cost-effective, like tACS. All the reviewed studies, which reported on the efficacy of tACS for neurocognitive disorders, suggested that tACS holds promise as a potential treatment for such conditions based on both neuropsychological assessments and neuroimaging metrics. However, due to the varied treatment parameters, determining the most effective tACS protocols remains challenging. The 40 Hz gamma frequency band, which was usually delivered at a peak-to-peak amplitude of 1-3 mA, was consistently applied for AD, dementia, and MCI (26–29, 36, 43, 46, 47, 49, 55, 75). The gamma frequency band has attracted attention in neurostimulation studies due to its crucial role in cognitive processes (81), in aligning bottom-up and top-down information, and in signifying the retrieval and application of that information (28). Thus, it is reasonable that gamma has been the predominant stimulation frequency in tACS studies for neurocognitive disorders, which manifest themselves as a deterioration from a previously achieved cognitive functioning level (81). Additionally, considering the crucial involvement of the frontal lobes, specifically the DLPFC, in various cognitive tasks like executive function, attention, memory, and language, it is unsurprising that this region has become a focal point in this field (82). The precuneus and temporal lobes were among the locations that also attracted interest for AD. Neuroimaging studies have demonstrated structural and functional alterations of the temporal lobe in AD (83, 84), while overactivation of the precuneus and its correlation with cognitive performance has been shown in pre-clinical stages of AD (85).

Two studies (46, 55) investigated the efficacy of a cumulative tACS program for MCI and dementia. Concurrent gamma-tACS and brain rehabilitation led to sustained positive changes in memory assessments up to 1-month follow-up, compared to only brain rehabilitation. Both studies had the same limitation of not including a sham condition to assess the placebo effects on post-stimulation cognitive improvements. Still, for an indication such as dementia, which inherently has cognitive deterioration, having no cognitive decline in addition to sustained or increased cognitive improvements at follow-up cannot be overlooked. At the same time, although a one-month follow-up could be informative for acute outcomes, the progressive nature of dementia necessitates a reassessment of these findings at extended follow-up durations. Moreover, the type of cognitive domains included in both the evaluation and the treatment protocol must be considered and accurately specified in future studies.

Beyond the documented cognitive enhancements, multiple studies on neurocognitive disorders reported alterations in spectral EEG and ERP components, as well as PET features, to provide a better understanding of the fundamental mechanisms of action of tACS. Gamma-tACS of the temporal lobes and precuneus led to an increase in gamma spectral power in target locations for AD (27, 28, 64). Gamma-tACS of the precuneus at the intensity of 3 mA also improved cognitive scores and enhanced SAI, an index of cholinergic transmission (27, 28). The treatment of AD used to be focused on cholinergic enhancement and reduction of beta-amyloid, but recent finding on aberrant gamma oscillations in AD highlights the need for a comprehensive approach to AD treatment (28). Notably, the precuneus and temporal areas are functionally linked, and the precuneus, as an accessible target for tACS, is one of the initial regions impacted in early-stage AD (27). Additionally, using perfusion-sensitive MRI scans, it has been demonstrated that brain perfusion (cerebral blood flow; CBF) increased significantly in the right temporal lobe following 10 sessions of gamma-tACS at the intensity of 4 mA over the right temporal lobe (64). Additionally, changes in CBF had a positive correlation with changes in cognitive performance as well as changes in spectral power of the gamma frequency band.

Of note is a study by Dhaynaut et al. (36), which administered 1 hour of daily gamma-tACS for 20 sessions, targeting the bi-temporal lobes in four participants with AD. Despite the absence of significant cognitive improvements, increases in gamma spectral power and decrease in p-Tau burden were shown after the treatment. Individualized and optimized stimulation parameters are required to detect significant behavioral changes as well as changes in neurobiological measures. Lastly, a comparison of the effects of gamma-tACS in patients with MCI and those with AD (56) showed that individuals with MCI who exhibited limited neuropsychological and electrophysiological changes akin to those observed in individuals with AD progressed to AD after a 2-year follow-up period. This finding supports the hypothesis that tACS could aid in identifying MCI patients who may be susceptible to dementia development, as well as the idea that tACS-EEG, like TMS-EEG, could provide perturbation-based biomarkers for early diagnosis and monitoring of disorders characterized by oscillopathies (86). While the reviewed studies consistently indicated the efficacy of tACS for addressing neurocognitive disorders with the above-mentioned parameters, establishing a biomarker for tracking the progression of neurocognitive disorders remains challenging. To enhance the reliability of these findings, future research should replicate these studies with larger sample sizes. Additionally, the results of a study (74) which found a reversion in cognitive scores at the 4-month follow-up after gamma-tACS of the temporal lobe necessitates the need for extended follow-up time points. Discussing the positive changes in cognitive skills following tACS in patients with neurocognitive disorders underscores the importance of studies on healthy populations. Research on healthy individuals helps to establish a baseline for normal physiological and cognitive functions, identify biomarkers of cognitive deficits, and allow for the meaningful generalization of findings (87). For instance, studies have shown that targeting specific brain regions with precise frequency modulation can selectively enhance memory functions in healthy older adults with memory impairments. Gamma-tACS applied to DLPFC significantly improved long-term memory, while theta-tACS applied to the inferior parietal lobule (IPL) enhanced working memory. Notably, switching the frequencies between DLPFC and IPL did not produce any memory benefits, highlighting the critical role of targeted frequency modulation (88).

With regards to the cognitive effects of tACS, it is essential to consider the impact of both retinal and somatosensory stimulations. Retinal phosphenes result from the spread of the current to the retina and can cause neural entrainment and thereby confound results (89). By affecting both perception and cognitive performance, phosphenes make it hard to differentiate between effects originating in the brain and those from the retina (89). Additionally, an alternative hypothesis suggests that tACS can also indirectly influence neural activities through peripheral sensory pathways, specifically by stimulating sensory fibers in the skin, and then delivering rhythmic signals to the central nervous system. Thus, defining the precise target location of tACS is challenging, as the affected areas would depend entirely on the somatosensory pathways activated by the skin near the electrodes (90). This further highlights the necessity of adding neuroimaging methods to tACS studies to learn more about its mechanisms of action and defining the optimized target location. Furthermore, it has been demonstrated that phase-tuned tACS can significantly improve cognitive performances in healthy subjects by aligning with endogenous brain oscillations (91, 92). On the other hand, the introduction of advanced algorithms like Stimulation Artifact Source Separation further enhances the precision of tACS by removing stimulation artifacts, enabling adaptive closed-loop systems (93). These findings underscore the importance of understanding the phase-dependent interactions between tACS and brain oscillations, suggesting that personalized stimulation protocols may offer superior outcomes over conventional methods.

Promising treatment outcomes for disorders with psychotic features were observed in the reviewed published studies which commonly targeted the DLPFC. A strong link between disrupted cortical oscillatory activity in the DLPFC and abnormalities in GABAergic functioning has consistently been observed in schizophrenia (41). Additionally, other types of neurostimulation (e.g., rTMS and tDCS) of the DLPFC have demonstrated potential for alleviating treatment-resistant negative symptoms of schizophrenia (44). Theta, as one of the most commonly applied stimulation frequencies for this indication (30, 44, 66, 67, 73), led to improvements in negative and/or positive symptoms of schizophrenia (30, 44, 73), as well as modulation of functional connectivity of the theta frequency band which related to negative symptoms of schizophrenia (73), and improvement in different cognitive skills (66, 67). Local and global increases in theta and delta power have been consistently observed in schizophrenia, however, cross-frequency coupling between gamma and theta, the impact of theta oscillations on gamma activity, and the role of this interaction in cognitive function need to be considered (94). When considering the published studies which applied gamma-tACS for psychotic disorders, it is evident that patients who received gamma-tACS had significant improvements in general psychopathology as well as negative and positive symptoms of schizophrenia and cognitive skills (39, 40). However, these studies had limited sample sizes and contradictory results should be considered. One study (41) did not find any significant changes in working memory assessments following gamma-tACS compared to tDCS and sham stimulation. This study applied gamma-tACS without an offset. Thus, introducing gamma-tACS with a direct current offset may be necessary for inducing excitability changes required to achieve gamma entrainment effectively. Apart from studies that confirmed safety and efficacy of tACS for neurocognitive disorders, it was also demonstrated that gamma-tACS in combination with cognitive training resulted in longer-lasting cognitive improvement than cognitive training alone, at the 1-month follow-up (46, 55). Both studies applied 40 Hz-gamma at the peak-to peak amplitude of 1.5 mA across 20 sessions, with each session lasting for 30 minutes. In contrast, a 4-month follow-up on cognitive improvement following gamma-tACS of the bilateral temporal lobes, with the same number of visits and same session duration, demonstrated that cognitive scores of one of the scales (ADAS) reverted to baseline levels, but sustained changes were observed in the MMSE scale (75).

A limited number of studies investigated the efficacy of alpha-tACS for auditory hallucinations in schizophrenia (24, 37, 52, 74). These studies applied alpha-tACS over either the DLPFC alone or DLPFC and the TPJ at 1-2 mA, with a different number and duration of sessions. Although alpha enhancement was shown following tACS (24, 74), only one study which applied alpha-tACS for ten 20-minutes sessions reported significant improvement in auditory hallucination (24). Additionally, the same duration and number of sessions caused a higher effect size for changes in auditory hallucination in alpha-tACS group compared to tDCS (52). These findings were aligned with previous studies demonstrating that alpha frequency band is correlated with negative symptoms of schizophrenia, and improvement of increased alpha amplitude following rTMS was associated with improvement of negative symptoms (94). At the same time, this highlights the importance of the duration of each tACS session and total number of stimulation visits to observe behavioral improvement along with changes in the imaging metrics. It also has been emphasized that the duration of visits has a crucial role in inducing neural plasticity which leads to tACS aftereffects (94).

Although these conflicting findings might imply that tACS has short-term efficacy, it also underlines the need for conducting clinical trials with personalized target locations based on imaging techniques, as well as the need to further investigate the role of the maintenance phase. Personalized tACS protocols consider the unique neurophysiological characteristics of each individual and can enhance treatment outcomes and optimize therapeutic interventions (95). One noteworthy conclusion from the sole study which compared delta-tACS to tDCS was that delta-tACS led to long-term positive changes while only neuroplastic changes of ERPs were observed in the tDCS group. This finding highlights the different mechanisms of action for different tES techniques regardless of having the same behavioral outcomes.

All studies that focused on conditions with depressive symptoms, except for one (57), reported improvements in either clinical, cognitive or imaging measures following tACS. The role of the frontal lobe in the neurobiology of depression should be highlighted, as the DLPFC followed by the bifrontal frontal cortices have been the most common tACS targets for depression. Various cognitive functions (96) and emotional regulation (97) originating from the frontal lobe may be associated with the development and manifestation of depression. Thus, targeting frontal areas with individualized treatment parameters could help to improve depressive symptoms. However, it is important to highlight that depression is a heterogeneous disorder. Its etiology involves the interplay of genetic, environmental, and neurobiological factors, and the precise mechanisms are still under investigation. It is also worth mentioning that although no predominant frequency band was reported in the ongoing clinical trials, alpha and gamma were the only applied stimulation frequencies in published studies. Patients with MDD show increased alpha band oscillatory activity, typically in the left frontal areas, and normalizing this activity helps to alleviate depressive symptoms (25). A single session of alpha-tACS was able to significantly reduce the power of left frontal alpha in both MDD (25, 61) and PMDD (60), and led to improvements of depressive symptoms in a patient with a lifelong history of depression (59). Additionally, in a 2-week follow-up after completion of frontal alpha-tACS and gamma-tACS, the alpha-tACS group had more responders compared with the gamma-tACS and sham groups (25). Reducing the strength of alpha asymmetry, a state of neuronal hypoactivity leading to disrupted affective processing, could potentially mitigate symptoms of depression (25). These findings consistently propose that left asymmetry of frontal alpha is a viable target for tACS studies on depression. Additionally, 40 Hz gamma-tACS at 2 mA intensity led to conflicting results for both cognitive and clinical symptoms of MDD. One study (40) which administered gamma-tACS to the bifrontal PFC with this protocol for either two daily 10-minutes sessions or a daily 20-minute session over ten days reported improvements in cognitive performance and clinical symptoms of MDD in both groups; however, the first group had better performance in both objective and subjective scales. Another study (57) administered a single session of tACS with this protocol over the left and right DLPFC for 20 minutes. This study demonstrated that a single session of gamma-tACS was not able to improve cognitive performance in MDD. However, the gamma frequency band has gained attention in neurostimulation studies for neuropsychiatric disorders (81). Interestingly, one study (68) with a larger sample size than the above mentioned studies revealed higher response and remission rates for drug-naive patients with MDD following 70 HZ gamma-tACS of the frontal and temporal cortices, at peak-to-peak amplitude of 15 mA over 20 40-minute sessions. Although it has been mentioned that gamma might be a potential biomarker of prefrontal dysfunction in MDD, and could be enhanced following antidepressant medications (57), the limited number of studies with small sample sizes and various treatment parameters precludes any conclusions on the efficacy of gamma-tACS for depression.

While most of the published studies had fewer than 20 sessions or even single-session tACS protocols, ongoing registered clinical trials tended to have protocols with higher number of sessions or with maintenance protocols. The approach of registered clinical trials is more consistent with the standard treatment regimen for rTMS which is daily sessions, up to 45 minutes, repeated five days a week for a course of 20–40 treatments (98). Moreover, the reviewed published studies have mostly investigated the acute effects of tACS and rarely included maintenance visits or long-term follow-ups. Additionally, it is important to note that while tACS has demonstrated good tolerability and safety across various studies, there remains a need for more extended follow-up to assess both the sustainability of positive changes and adverse events associated with tACS in the long-term. It is also crucial to consider the association between the intensity of tACS with its safety and tolerability. As tACS is applied transcutaneously, it can become intolerable at higher intensities; increased amplitudes are associated with increased side effects such as burning, dizziness, phosphenes, and metallic taste (99). However, increasing the amplitude also creates a stronger electric field in the brain, leading to greater membrane depolarization and spike entrainment, and thus, possibly larger behavioral effects (100). As such, in determining the optimal amplitude for tACS, it is important to consider the tradeoff between the increased magnitude of effects and increased side effects associated with greater amplitudes. Ensuring the safety of tACS requires ongoing vigilance in reviewing the literature, given the limited number of available reports addressing potential adverse events linked to this technique (101).

The reviewed literature illustrates that tACS shows promise as an interventional technique for neuropsychiatric disorders. Nevertheless, within the realm of clinical trials, numerous questions remain regarding the sustained “offline” effects on network dynamics following tACS application, the underlying mechanisms of action of tACS, and the optimal treatment parameters (15). Moreover, one important factor regarding outcome measurement which was overlooked in many of the reviewed published studies was the state-dependence of tACS. Recording neural oscillations before and after the intervention is crucial to individualize treatment parameters, ascertain the engagement of the desired target, and distinguish whether changes in EEG are genuinely attributed to tACS. Thus, it is essential to recognize that while tACS shows promise in neuropsychiatric research, it is not yet a standard clinical treatment for these disorders, and the field continues to evolve.

## 5 Conclusion

Altered gamma oscillations, particularly in the frontal areas of the brain, have been repeatedly reported as potential biomarkers of both emotional- and cognitive-related symptoms of neuropsychiatric disorders, and this systematic review also showed that gamma-tACS of the DLPFC, along with other frontal sites, has been the most common modality in tACS studies. However, different study designs and tACS protocols (i.e., number of sessions, duration of each session, number of daily sessions, and stimulation montage) make it difficult to draw conclusions on the optimal parameters for each indication. Additionally, contradictory results from the same stimulation parameters highlight the need for moving toward more personalized treatment parameters based on neuroimaging techniques. While EEG metrics consistently showed that tACS can enhance the EEG power spectrum around the stimulation frequency in targeted locations, the majority of studies did not investigate the neurophysiological aspects of tACS. Thus, as tACS has shown promise for modulating existing and ongoing neural oscillations in many conditions, more prospective biomarker-focused studies are expected to characterize its relationship with treatment response. Research efforts in this direction could help develop personalized treatment protocols, which have the potential to be delivered in at-home settings.

Several challenges restrict tACS from becoming a first-line treatment for neuropsychiatric disorders. Consistent evidence of clinical efficacy and standardized treatment protocols is lacking, and the mechanisms of action for tACS remain poorly understood. Existing studies vary in methodology, design, and stimulation parameters, and a better understanding of tACS mechanisms through functional and structural neuroimaging techniques is required. It is important to note that while tACS is generally safe and well-tolerated, comprehensive long-term safety data are still required, as most current studies only report short-term safety outcomes.

## Data Availability

The data that support the findings of this study are available from the corresponding author upon reasonable request.
