# Supplementary material for: Transcranial alternating current stimulation for neuropsychiatric disorders: a systematic review of treatment parameters and outcomes
Source: Front Psychiatry. 2024 Aug 14;15:1419243. doi: 10.3389/fpsyt.2024.1419243 (PMC11360874; doi:10.3389/fpsyt.2024.1419243)
Supplement: Supplementary file 1 [file DataSheet1.docx]

**Transcranial Alternating Current Stimulation for Neuropsychiatric Disorders: A Systematic Review of Treatment Parameters and Outcomes**

***Supplementary Data***

**Supplementary Table 1a.** PRISMA 2020 for Abstracts Checklist.

| **Section and Topic** | **Item #** | **Checklist item** | **Reported (Yes/No)** |
| --- | --- | --- | --- |
| **TITLE** | | |  |
| Title | 1 | Identify the report as a systematic review. | Yes |
| **BACKGROUND** | | |  |
| Objectives | 2 | Provide an explicit statement of the main objective(s) or question(s) the review addresses. | Yes |
| **METHODS** | | |  |
| Eligibility criteria | 3 | Specify the inclusion and exclusion criteria for the review. | Yes |
| Information sources | 4 | Specify the information sources (e.g. databases, registers) used to identify studies and the date when each was last searched. | Yes |
| Risk of bias | 5 | Specify the methods used to assess risk of bias in the included studies. | Yes |
| Synthesis of results | 6 | Specify the methods used to present and synthesise results. | N/A |
| **RESULTS** | | |  |
| Included studies | 7 | Give the total number of included studies and participants and summarise relevant characteristics of studies. | Yes |
| Synthesis of results | 8 | Present results for main outcomes, preferably indicating the number of included studies and participants for each. If meta-analysis was done, report the summary estimate and confidence/credible interval. If comparing groups, indicate the direction of the effect (i.e. which group is favoured). | Yes |
| **DISCUSSION** | | |  |
| Limitations of evidence | 9 | Provide a brief summary of the limitations of the evidence included in the review (e.g. study risk of bias, inconsistency and imprecision). | Yes |
| Interpretation | 10 | Provide a general interpretation of the results and important implications. | Yes |
| **OTHER** | | |  |
| Funding | 11 | Specify the primary source of funding for the review. | N/A |
| Registration | 12 | Provide the register name and registration number. | N/A |

*From:*  Page MJ, McKenzie JE, Bossuyt PM, Boutron I, Hoffmann TC, Mulrow CD, et al. The PRISMA 2020 statement: an updated guideline for reporting systematic reviews. BMJ 2021;372:n71. doi: 10.1136/bmj.n71

For more information, visit: <http://www.prisma-statement.org/>

**Supplementary Table 1b.** PRISMA 2020 Checklist.

| **Section and Topic** | **Item #** | **Checklist item** | **Location where item is reported** |
| --- | --- | --- | --- |
| **TITLE** | | |  |
| Title | 1 | Identify the report as a systematic review. | Title page |
| **ABSTRACT** | | |  |
| Abstract | 2 | See the PRISMA 2020 for Abstracts checklist. | Supplementary Table 1a |
| **INTRODUCTION** | | |  |
| Rationale | 3 | Describe the rationale for the review in the context of existing knowledge. | Introduction, para. 1-4 |
| Objectives | 4 | Provide an explicit statement of the objective(s) or question(s) the review addresses. | Introduction, para. 3 |
| **METHODS** | | |  |
| Eligibility criteria | 5 | Specify the inclusion and exclusion criteria for the review and how studies were grouped for the syntheses. | Methods > 2.2 Inclusion and exclusion criteria  Supplementary Table 3 |
| Information sources | 6 | Specify all databases, registers, websites, organisations, reference lists and other sources searched or consulted to identify studies. Specify the date when each source was last searched or consulted. | Methods > 2.1 Search strategy |
| Search strategy | 7 | Present the full search strategies for all databases, registers and websites, including any filters and limits used. | Supplementary Table 2 |
| Selection process | 8 | Specify the methods used to decide whether a study met the inclusion criteria of the review, including how many reviewers screened each record and each report retrieved, whether they worked independently, and if applicable, details of automation tools used in the process. | Methods > 2.2 Inclusion and exclusion criteria  Supplementary Table 3 |
| Data collection process | 9 | Specify the methods used to collect data from reports, including how many reviewers collected data from each report, whether they worked independently, any processes for obtaining or confirming data from study investigators, and if applicable, details of automation tools used in the process. | Methods >2.2 Inclusion and exclusion criteria |
| Data items | 10a | List and define all outcomes for which data were sought. Specify whether all results that were compatible with each outcome domain in each study were sought (e.g. for all measures, time points, analyses), and if not, the methods used to decide which results to collect. | Methods > 2.3 Variable extraction |
|  | 10b | List and define all other variables for which data were sought (e.g. participant and intervention characteristics, funding sources). Describe any assumptions made about any missing or unclear information. | Methods > 2.3 Variable extraction |
| Study risk of bias assessment | 11 | Specify the methods used to assess risk of bias in the included studies, including details of the tool(s) used, how many reviewers assessed each study and whether they worked independently, and if applicable, details of automation tools used in the process. | Methods > 2.4 Assessment of quality for published literature  Supplementary Table 5 |
| Effect measures | 12 | Specify for each outcome the effect measure(s) (e.g. risk ratio, mean difference) used in the synthesis or presentation of results. | N/A |
| Synthesis methods | 13a | Describe the processes used to decide which studies were eligible for each synthesis (e.g. tabulating the study intervention characteristics and comparing against the planned groups for each synthesis (item #5)). | Methods > 2.2 Inclusion and exclusion criteria &  Supplementary Table 3 |
|  | 13b | Describe any methods required to prepare the data for presentation or synthesis, such as handling of missing summary statistics, or data conversions. | N/A |
|  | 13c | Describe any methods used to tabulate or visually display results of individual studies and syntheses. | N/A |
|  | 13d | Describe any methods used to synthesize results and provide a rationale for the choice(s). If meta-analysis was performed, describe the model(s), method(s) to identify the presence and extent of statistical heterogeneity, and software package(s) used. | Methods > 2.3 Variable extraction |
|  | 13e | Describe any methods used to explore possible causes of heterogeneity among study results (e.g. subgroup analysis, meta-regression). | N/A |
|  | 13f | Describe any sensitivity analyses conducted to assess robustness of the synthesized results. | N/A |
| Reporting bias assessment | 14 | Describe any methods used to assess risk of bias due to missing results in a synthesis (arising from reporting biases). | N/A |
| Certainty assessment | 15 | Describe any methods used to assess certainty (or confidence) in the body of evidence for an outcome. | Table 1 |
| **RESULTS** | | |  |
| Study selection | 16a | Describe the results of the search and selection process, from the number of records identified in the search to the number of studies included in the review, ideally using a flow diagram. | Results > Results &  Figure 1 |
|  | 16b | Cite studies that might appear to meet the inclusion criteria, but which were excluded, and explain why they were excluded. | Supplementary Table 4 |
| Study characteristics | 17 | Cite each included study and present its characteristics. | Table 1 |
| Risk of bias in studies | 18 | Present assessments of risk of bias for each included study. | Results > 3.1 Quality assessment &  Supplementary Table 5 |
| Results of individual studies | 19 | For all outcomes, present, for each study: (a) summary statistics for each group (where appropriate) and (b) an effect estimate and its precision (e.g. confidence/credible interval), ideally using structured tables or plots. | Table 1 |
| Results of syntheses | 20a | For each synthesis, briefly summarise the characteristics and risk of bias among contributing studies. | *Characteristics*: Results, Table 1  *Risk of bias***:** Supplementary Table 5 |
|  | 20b | Present results of all statistical syntheses conducted. If meta-analysis was done, present for each the summary estimate and its precision (e.g. confidence/credible interval) and measures of statistical heterogeneity. If comparing groups, describe the direction of the effect. | N/A |
|  | 20c | Present results of all investigations of possible causes of heterogeneity among study results. | Results (all paras.) |
|  | 20d | Present results of all sensitivity analyses conducted to assess the robustness of the synthesized results. | N/A |
| Reporting biases | 21 | Present assessments of risk of bias due to missing results (arising from reporting biases) for each synthesis assessed. | N/A |
| Certainty of evidence | 22 | Present assessments of certainty (or confidence) in the body of evidence for each outcome assessed. | Table 1 |
| **DISCUSSION** | | |  |
| Discussion | 23a | Provide a general interpretation of the results in the context of other evidence. | Discussion para. 2-12 |
|  | 23b | Discuss any limitations of the evidence included in the review. | Discussion para. 13 |
|  | 23c | Discuss any limitations of the review processes used. | Discussion para. 13 |
|  | 23d | Discuss implications of the results for practice, policy, and future research. | Conclusion |
| **OTHER INFORMATION** | | |  |
| Registration and protocol | 24a | Provide registration information for the review, including register name and registration number, or state that the review was not registered. | N/A |
|  | 24b | Indicate where the review protocol can be accessed, or state that a protocol was not prepared. | N/A |
|  | 24c | Describe and explain any amendments to information provided at registration or in the protocol. | N/A |
| Support | 25 | Describe sources of financial or non-financial support for the review, and the role of the funders or sponsors in the review. | Funding |
| Competing interests | 26 | Declare any competing interests of review authors. | Conflict of Interest |
| Availability of data, code and other materials | 27 | Report which of the following are publicly available and where they can be found: template data collection forms; data extracted from included studies; data used for all analyses; analytic code; any other materials used in the review. | Data Availability Statement |

*From:*  Page MJ, McKenzie JE, Bossuyt PM, Boutron I, Hoffmann TC, Mulrow CD, et al. The PRISMA 2020 statement: an updated guideline for reporting systematic reviews. BMJ 2021;372:n71. doi: 10.1136/bmj.n71

For more information, visit: <http://www.prisma-statement.org/>

**Supplementary Table 2a.** OVID search strategy.

Database(s): **Embase Classic+Embase**1947 to 2023 May 03**, APA PsycInfo**1806 to April Week 4 2023**, Ovid MEDLINE(R) ALL**1946 to May 03, 2023

Search Strategy:

| **#** | **Searches** | **Results** |
| --- | --- | --- |
| 1 | exp mental disorders/ | 5219589 |
| 2 | ((mental or psychiatric or psychological or neurodevelopmental or neurocognitive or neuropsychiatric or trauma* or neuropsychological) adj3 (diagnos#s or disorder* or disease* or illness* or condition*)).tw,kf,id. | 904269 |
| 3 | (anxiety or agoraphobia or phobia or panic or phobic or mutism).tw,kf,id. | 922455 |
| 4 | (trauma-related disorder* or social engagement disorder* or attachment disorder* or adjustment disorder* or stress disorder* or posttraumatic stress disorder* or post traumatic stress disorder* or post-traumatic stress disorder* or PTSD).tw,kf,id. | 167376 |
| 5 | (trichotillomani* or excoriation or skin picking disorder* or skin-picking disorder* or hair-pulling disorder* or hair pulling disorder* or hoarding disorder* or body dysmorphi* or dysmorphic disorder* or obsessive compulsive disorder* or obsessive-compulsive disorder* or OCD).tw,kf,id. | 75365 |
| 6 | (rumination disorder* or binge eating disorder* or binge-eating disorder* or anorexi* or bulimi* or orthorexi* or pica or feeding disorder* or eating disorder*).tw,kf,id. | 195480 |
| 7 | (elimination disorder* or enures#s or encopres#s).tw,kf,id. | 17338 |
| 8 | (bipolarity or mania or hypomania or manic or hypomanic or cyclothymic or cyclothymia).tw,kf,id. | 81339 |
| 9 | ((bipolar or mood or affect* or depressive) adj3 (disorder* or illness*)).tw,kf,id. | 472289 |
| 10 | (major depressi* or dysthymi*).tw,kf,id. | 199811 |
| 11 | (MDD or TRD).tw,kf,id. | 65623 |
| 12 | ((treatment resistant or treatment-resistant) adj2 (depression or bipolar)).tw,kf,id. | 13490 |
| 13 | (catatoni* or schizoaffective or schizophreni* or psychos#s or psychotic or hallucination* or delusion* or schizotyp*).tw,kf,id. | 718963 |
| 14 | ((borderline or antisocial or histrionic or narcissistic or schizoid or schizotypal or paranoi* or delusion* or compulsive or obsessive-compulsive or passive-aggressive or avoidant or dependent) adj3 (disorder* or illness*)).tw,kf,id. | 123820 |
| 15 | (psychopath* or sociopath*).tw,kf,id. | 224236 |
| 16 | (explosive disorder* or pyromani* or kleptomani* or conduct disorder* or defiant disorder* or gambling disorder* or pathological gambling).tw,kf,id. | 38199 |
| 17 | (dissociative disorder* or dissociative amnesi* or dissociative fugue* or identity disorder* or depersonalization* or derealization* or fugue state*).tw,kf,id. | 22661 |
| 18 | exp Substance-Related Disorders/ or exp "substance use disorder"/ or exp addiction/ | 824016 |
| 19 | ((substance or drug or sedative* or stimulant* or alcohol* or hallucinogen* or phencyclidine or inhalant* or caffeine or opioid or cocaine or amphetamine or cannabis or marijuana or nicotine or tobacco) adj3 (dependence or abuse or addiction* or withdrawal)).tw,kf,id. | 423522 |
| 20 | ((substance or drug or sedative* or stimulant* or alcohol* or hallucinogen* or phencyclidine or inhalant* or caffeine or opioid or cocaine or amphetamine or cannabis or marijuana or nicotine or tobacco) adj2 disorder*).tw,kf,id. | 175134 |
| 21 | alcoholism.tw,kf,id. | 100732 |
| 22 | ((addiction or addictive) adj disorder*).tw,kf,id. | 7443 |
| 23 | exp dementia/ | 742555 |
| 24 | neurocognitive disorder*.tw,kf,id. | 12575 |
| 25 | (alzheimer* or delirium or lewy bod* or mild cognitive impairment).tw,kf,id. | 626441 |
| 26 | (suicidality or suicide or suicidal).tw,kf,id. | 293662 |
| 27 | anhedoni*.tw,kf,id. | 19874 |
| 28 | exp cognitive impairment/ or exp neurobehavioral manifestations/ | 1469745 |
| 29 | anhedonia/ or catatonia/ or dyslexia/ or language development disorders/ or echolalia/ or mutism/ or stuttering/ or learning disabilities/ or dyscalculia/ or amnesia/ | 204925 |
| 30 | (autism* or Asperger*).tw,kf,id. | 203969 |
| 31 | ADHD.tw,kf,id. | 111011 |
| 32 | (attention deficit hyperactivity or attention-deficit-hyperactivity).tw,kf,id. | 103265 |
| 33 | attention deficit disorder*.tw,kf,id. | 10821 |
| 34 | hyperactivity disorder*.tw,kf,id. | 104578 |
| 35 | (dyslexi* or dysgraph* or language development* disorder* or intellectual disabilit* or intellectual development* disorder* or echolali* or stuttering or learning disabilit* or dyscalculi* or amnesi* or mental retard* or intellectual development* delay or language disorder* or speech disorder* or speech sound disorder* or phonological disorder* or fluency disorder* or communication disorder* or learning disorder* or coordination disorder* or stereotyp*).tw,kf,id. | 440674 |
| 36 | exp tic disorders/ | 27073 |
| 37 | (tourette* or tic disorder*).tw,kf,id. | 21630 |
| 38 | Tourette Syndrome/ | 15548 |
| 39 | (factitious disorder* or FNSD or functional neurological syndrome disorder* or conversion disorder* or illness anxiety or hypochondria* or somatic symptom disorder* or somatic disorder*).tw,kf,id. | 24755 |
| 40 | (gender dysphori* or gender identity disorder*).tw,kf,id. | 8243 |
| 41 | (transvesti* or fetishis* or sadis* or masochis* or paraphili* or pedophili* or voyeuris* or exhibitionis* or frotteuris*).tw,kf,id. | 18921 |
| 42 | exp Sleep Wake Disorders/ | 439284 |
| 43 | (sleep terror* or night terror* or sleep arousal disorder* or sleepwalking or nightmare* or hypersomni* or parasomni* or sleep wake disorder* or somnambul* or insomnia* or hypersomnolen* or narcolep*).tw,kf,id. | 134109 |
| 44 | (huntington* or parkinson*).tw,kf,id. | 443026 |
| 45 | Huntington Disease/ | 39328 |
| 46 | Parkinson Disease/ | 275032 |
| 47 | Lewy Body Disease/ | 14413 |
| 48 | or/1-47 | 7523273 |
| 49 | alternat* current stimulation*.tw,kf,id. | 2695 |
| 50 | tACS.tw,kf,id. | 5436 |
| 51 | (stimulation adj3 alternat* current*).tw,kf,id. | 2876 |
| 52 | or/49-51 | 6098 |
| 53 | 48 and 52 | 1171 |
| 54 | remove duplicates from 53 | 783 |

**Supplementary Table 2b.** Search strategy for clinical trials.

Total clinical trials [800 from Clinicaltrials.gov + 227 from ICTRP] = 1027

Total clinical trials after removing duplicates = 373

| **Clinicaltrials.gov: Advanced search** | | |
| --- | --- | --- |
| **Results** | **Condition or Disease** | **Intervention/treatment** |
| 88 | mental disorder OR mental disease OR mental diagnosis OR mental illness OR mental condition | alternating current stimulation OR tACS |
| 88 | psychiatric disorder OR psychiatric disease OR psychiatric diagnosis OR psychiatric illness OR psychiatric condition |  |
| 90 | psychological disorder OR psychological disease OR psychological diagnosis OR psychological illness OR psychological condition |  |
| 6 | neurodevelopmental disorder OR neurodevelopmental disease OR neurodevelopmental diagnosis OR neurodevelopmental illness OR neurodevelopmental condition |  |
| 39 | neurocognitive disorder OR neurocognitive disease OR neurocognitive diagnosis OR neurocognitive illness OR neurocognitive condition |  |
| 0 | neuropsychiatric disorder OR neuropsychiatric disease OR neuropsychiatric diagnosis OR neuropsychiatric illness OR neuropsychiatric condition |  |
| 0 | neuropsychological disorder OR neuropsychological disease OR neuropsychological diagnosis OR neuropsychological illness OR neuropsychological condition |  |
| 71 | trauma |  |
| 6 | anxiety OR agoraphobia OR phobia OR panic OR phobic OR mutism |  |
| 3 | trauma-related disorder OR social engagement disorder OR attachment disorder OR adjustment disorder OR stress disorder OR posttraumatic stress disorder OR post traumatic stress disorder OR post-traumatic stress disorder OR PTSD |  |
| 0 | trichotillomania OR excoriation OR skin picking disorder OR skin-picking disorder OR hair-pulling disorder OR hair pulling disorder |  |
| 0 | hoarding disorder OR body dysmorphia OR dysmorphic disorder OR obsessive compulsive disorder OR obsessive-compulsive disorder OR OCD |  |
| 1 | rumination disorder OR binge eating disorder OR binge-eating disorder OR anorexia OR bulimia OR prothorax OR pica OR feeding disorder OR eating disorder |  |
| 1 | bipolarity OR mania OR hypomania OR manic OR hypomanic OR cyclothymic OR cyclothymia |  |
| 4 | bipolar disorder OR bipolar symptom OR bipolar illness |  |
| 12 | mood disorder OR mood symptom OR mood illness |  |
| 12 | affect disorder OR affect symptom OR affect illness |  |
| 11 | depressive disorder OR depressive symptom OR depressive illness |  |
| 6 | major depressive OR major depression OR dysthymia |  |
| 7 | MDD OR TRD |  |
| 0 | treatment resistant depression OR treatment-resistant depression |  |
| 0 | treatment resistant bipolar OR treatment-resistant bipolar |  |
| 88 | catatonia OR schizoaffective OR schizophrenia OR psychosis OR psychotic or hallucination OR delusion OR schizotypal |  |
| 1 | borderline disorder OR antisocial disorder OR histrionic disorder OR narcissistic disorder OR schizoid disorder OR schizotypal disorder OR paranoia disorder |  |
| 2 | delusion disorder OR compulsive disorder OR obsessive-compulsive disorder OR passive-aggressive disorder OR avoidant disorder OR dependent disorder |  |
| 0 | borderline symptom OR antisocial symptom OR histrionic symptom OR narcissistic symptom OR schizoid symptom OR schizotypal symptom OR paranoia symptom |  |
| 1 | delusion symptom OR compulsive symptom OR obsessive-compulsive symptom OR passive-aggressive symptom OR avoidant symptom OR dependent symptom |  |
| 0 | borderline illness OR antisocial illness OR histrionic illness OR narcissistic illness OR schizoid illness OR schizotypal illness OR paranoia illness |  |
| 0 | delusion illness OR compulsive illness OR obsessive-compulsive illness OR passive-aggressive illness OR avoidant illness OR dependent illness |  |
| 0 | psychopath OR sociopath |  |
| 8 | explosive disorder OR pyromania OR kleptomania OR conduct disorder OR defiant disorder OR gambling disorder OR pathological gambling |  |
| 4 | dissociative disorder OR dissociative amnesia OR dissociative fugue OR identity disorder OR depersonalization OR derealization OR fugue state |  |
| 5 | Substance-Related Disorders |  |
| 2 | substance dependence OR drug dependence OR sedative dependence OR stimulant dependence OR alcohol dependence OR hallucinogen dependence OR phencyclidine dependence OR inhalant dependence |  |
| 0 | caffeine dependence OR opioid dependence OR cocaine dependence OR amphetamine dependence OR cannabis dependence OR marijuana dependence OR nicotine dependence OR tobacco dependence |  |
| 0 | substance abuse OR drug abuse OR sedative abuse OR stimulant abuse OR alcohol abuse OR hallucinogen abuse OR phencyclidine abuse OR inhalant abuse |  |
| 0 | caffeine abuse OR opioid abuse OR cocaine abuse OR amphetamine abuse OR cannabis abuse OR marijuana abuse OR nicotine abuse OR tobacco abuse |  |
| 2 | substance addiction OR drug addiction OR sedative addiction OR stimulant addiction OR alcohol addiction OR hallucinogen addiction OR phencyclidine addiction OR inhalant addiction |  |
| 0 | caffeine addiction OR opioid addiction OR cocaine addiction OR amphetamine addiction OR cannabis addiction OR marijuana addiction OR nicotine addiction OR tobacco addiction |  |
| 3 | substance withdrawal OR drug withdrawal OR sedative withdrawal OR stimulant withdrawal OR alcohol withdrawal OR hallucinogen withdrawal OR phencyclidine withdrawal OR inhalant withdrawal |  |
| 1 | caffeine withdrawal OR opioid withdrawal OR cocaine withdrawal OR amphetamine withdrawal OR cannabis withdrawal OR marijuana withdrawal OR nicotine withdrawal OR tobacco withdrawal |  |
| 43 | substance disorder OR drug disorder OR sedative disorder OR stimulant disorder OR alcohol disorder OR hallucinogen disorder OR phencyclidine disorder OR inhalant disorder |  |
| 4 | caffeine disorder OR opioid disorder OR cocaine disorder OR amphetamine disorder OR cannabis disorder OR marijuana disorder OR nicotine disorder OR tobacco disorder |  |
| 1 | alcoholism |  |
| 0 | Addiction disorder OR addictive disorder |  |
| 21 | dementia |  |
| 39 | neurocognitive disorder |  |
| 32 | Alzheimer’s OR delirium OR Lewy body OR mild cognitive impairment |  |
| 1 | suicidality OR suicide OR suicidal |  |
| 1 | anhedonia |  |
| 33 | neurobehavioral manifestations |  |
| 7 | anhedonia OR catatonia OR dyslexia OR language development disorders OR echolalia OR mutism OR stuttering OR learning disabilities OR dyscalculia OR amnesia |  |
| 2 | autism OR Asperger |  |
| 1 | ADHD |  |
| 0 | attention deficit hyperactivity OR attention-deficit-hyperactivity |  |
| 1 | attention deficit disorder |  |
| 1 | hyperactivity disorder |  |
| 3 | dyslexia OR dysgraphia OR language development disorder OR intellectual disability OR intellectual disabilities OR intellectual development disorder OR echolalia OR stuttering OR learning disability |  |
| 13 | learning disabilities OR dyscalculia OR amnesia OR mental retard OR intellectual development delay OR language disorder OR speech disorder OR speech sound disorder OR phonological disorder |  |
| 11 | fluency disorder OR communication disorder OR learning disorder OR coordination disorder OR stereotype |  |
| 0 | tic disorders |  |
| 0 | Tourette OR tic disorder |  |
| 0 | Tourette Syndrome |  |
| 4 | factitious disorder OR FNSD OR functional neurological syndrome disorder OR conversion disorder OR illness anxiety OR hypochondria OR somatic symptom disorder OR somatic disorder |  |
| 0 | gender dysphoria OR gender identity disorder |  |
| 0 | transvestic OR fetishes OR sadism OR masochism OR paraphilia OR pedophilia OR voyeurism OR exhibitionism OR frotteurs |  |
| 3 | Sleep Wake Disorders OR Sleep-Wake Disorders |  |
| 5 | sleep terror OR night terror OR sleep arousal disorder OR sleepwalking OR nightmare OR hypersomnia OR parasomnia OR sleep wake disorder OR somnambule OR insomnia OR hypersomnia OR narcolepsy |  |
| 5 | Huntington OR Parkinson |  |
| 0 | Huntington Disease |  |
| 5 | Parkinson Disease |  |
| 2 | Lewy Body Disease |  |
| **ICTRP: general search** | | |
| **Results** | **Condition or Disease** | **Intervention/treatment** |
| 227 | Unspecified | alternating current stimulation OR tACS |

**Supplementary Table 3.** Eligibility criteria.

| ***Inclusion criteria*** |
| --- |
| Include a study if:   - At least one of the study groups includes one of the neuropsychiatric disorders listed in the APA DSM IV and DSM-5. Participants with two or more psychiatric comorbidities are allowed, as long as the comorbid condition is listed in the DSM IV or DSM-5. - At least one of the study arms has received verum tACS delivered to the scalp. tACS in combination with other treatments, either in one arm or separate arms, are allowed but should be clearly noted. - tACS is administered as an intervention in a clinical study setting. - It falls in any of these categories: case reports, case series, letters, editorials, notes, preprints, errata/corrigenda, conference original research papers, protocol papers, unpublished clinical trials from CT.gov and ICTRP. - Protocol papers will be categorized and reviewed as published studies. - If a registered clinical trial is associated with a publication, the publication should be located, and the study will be categorized and reviewed as a published study. - As a published paper, it is an original published research study (journal articles). - Participants are aged 18+ . - It is in English. - It has human subjects. |
| ***Exclusion criteria*** |
| Exclude a study if:   - It falls in any of these categories: systematic reviews, books, chapters, theses/dissertations, conference abstracts, conference reviews, meta-analyses - None of the assessed tACS effects are related to clinical, behavioral, cognitive, neurophysiological, and electrophysiological measures. Studies on computational modelling of tACS effects, without reporting one of the above mentioned outcomes, should be excluded. - Participants have neuropsychiatric symptoms caused by medical, organic or drug-related factors. |

**Supplementary Table 4a.** Excluded published studies in the second level screening.

|  | **Citation** | **Reason for exclusion** |
| --- | --- | --- |
| 1 | Klimke. (2016). Case Report: Successful Treatment of Therapy-Resistant OCD with Application of Transcranial Alternating Current Stimulation (tACS). Brain Stimulation., 9(3), 463–465. https://doi.org/10.1016/j.brs.2016.03.005 | Wrong patient population (OCD) |
| 2 | Esmat, N. (1975). Electro-therapy instead of sedatives, tranquilizers, and hypnotics by alternating current stimulation “electro-relaxation”. Journal of the Egyptian Medical Association, 58(3–4), 234–241. https://doi.org/info:doi/ | Report not retrieved |
| 3 | Wechselstromstimulation als Ersttherapie bei Depression. (2022). Fortschritte Der Neurologie, Psychiatrie., 90(12), 553. https://doi.org/10.1055/a-1832-0020 | Wrong language  (non-English) |
| 4 | McAleer, J., McAleer, L., Stewart, R., Shepard, M., Sheena, S., Kabir, I., Swank, J. P., Stange, A., Leow, H., & Klumpp, O. (2023). Differential effects of transcranial current type on heart rate variability during emotion regulation in internalizing psychopathologies. Journal of Affective Disorders., 327, 7–14. https://doi.org/10.1016/j.jad.2023.01.102 | Wrong language  (non-English) |
| 5 | Wang, H. X., Wang, K., Sun, Z. C., Peng, M., Xue, Q., Li, N., ... & Wang, Y. P. (2020). A pilot study of transcranial alternating current stimulation in the treatment of drug-naive adult patients with major depressive disorder. Zhonghua yi xue za zhi, 100(3), 197-201. | Wrong language  (non-English) |
| 6 | Haller. (2020). Gamma transcranial alternating current stimulation (gammatACS) in obsessive-compulsive disorder: A case report. Fortschritte Der Neurologie, Psychiatrie., 88(6), 398–401. https://doi.org/info:doi/ | Wrong language  (non-English) |
| 7 | Amouzadeh, F. (2022). Impact of transcranial alternating current stimulation on working memory and selective attention in athletes with attention deficit hyperactivity disorder: randomized controlled trial. NeuroReport, 33(17), 756-762. | Wrong language  (non-English) |
| 8 | Davis. (2022). Medial prefrontal transcranial alternating current stimulation for apathy in Huntington’s disease. MedRxiv. https://doi.org/10.1101/2022.08.29.22279310 | Data copy |
| 9 | Thirugnanasambandam, N., Kasten, F. H., & Udupa, K. (2021). Novel Multimodal Approaches in Non-invasive Brain Stimulation. Frontiers in Human Neuroscience, 15, 784637. | Ineligible study type  (Editorial article) |
| 10 | Frohlich, F., Frohlich, J., & Riddle, J. S. (2021). Transcranial alternating current stimulation for the treatment of obsessive-compulsive disorder? Brain Stimulation, 14(4), 1048–1050. https://doi.org/10.1016/j.brs.2021.06.014 | Insufficient data |
| 11 | Teo. (2017). Transcranial alternating current stimulation: A potential modulator for pathological oscillations in Parkinson’s disease? Frontiers in Neurology., 8(MAY). https://doi.org/10.3389/fneur.2017.00185 | Ineligible study type  (Review paper) |
| 12 | Walia. (2022). Portable Neuroimaging-Guided Noninvasive Brain Stimulation of the Cortico-Cerebello-Thalamo-Cortical Loop-Hypothesis and Theory in Cannabis Use Disorder. Brain Sciences., 12(4). https://doi.org/10.3390/brainsci12040445 | Wrong patient population (healthy subjects) |
| 13 | Clancy, K. J., Clancy, S. K., Baisley, A., Albizu, N., Kartvelishvili, M., & Ding, W. (2018). Lasting connectivity increase and anxiety reduction via transcranial alternating current stimulation. Social Cognitive and Affective Neuroscience., 13(12), 1305–1316. https://doi.org/10.1093/scan/nsy096 | Wrong patient population  (healthy subjects) |
| 14 | Varastegan, S., Varastegan, R., Kazemi, R., Rostami, S., Khomami, A., & Zandbagleh, A. L. (2023). Remember NIBS? tACS improves memory performance in elders with subjective memory complaints. GeroScience., 45(2), 851–869. https://doi.org/10.1007/s11357-022-00677-2 | Wrong patient population  (Memory complaints) |
| 15 | Greenwald. (2022). A randomized, sham-controlled, quintuple-blinded trial to evaluate the NET device as an alternative to medication for promoting opioid abstinence. Contemporary Clinical Trials Communications, 30. https://doi.org/10.1016/j.conctc.2022.101018 | Ineligible topic  NeuroElectric Therapy |
| 16 | Reinhart, R. M. G. (2017). Disruption and rescue of interareal theta phase coupling and adaptive behavior. Proceedings of the National Academy of Sciences of the United States of America., 114(43), 11542–11547. https://doi.org/10.1073/pnas.1710257114 | Wrong patient population  (healthy subjects) |
| 17 | Corrigendum: Protocol on transcranial alternating current stimulation for the treatment of major depressive disorder: a randomized controlled trial. (2020). Chinese Medical Journal., 133(8), 1008. https://doi.org/10.1097/CM9.0000000000000763 | Data copy |
| 18 | McKim, T. H., McKim, S. J., Dove, D. L., Robinson, F., & Fröhlich, C. A. (2021). Addiction history moderates the effect of prefrontal 10-Hz transcranial alternating current stimulation on habitual action selection. Journal of Neurophysiology /, 125(3), 768–780. https://doi.org/10.1152/JN.00180.2020 | Data copy |
| 19 | Wang, H.-X., Wang, K., Wang, W.-R., Zhang, W.-F., Zhao, X.-T., Yang, L., Wang, M., Peng, Z.-C., Sun, Q., Xue, Y., Jia, N., Li, K., Dong, Q., Zhang, S.-Q., Zhan, B.-Q., Min, C.-Q., Fan, A.-H., Zhou, H.-Q., Song, L., … Si, J. (2020). Protocol on transcranial alternating current stimulation for the treatment of major depressive disorder: a randomized controlled trial. Chinese Medical Journal., 133(1), 61–67. https://doi.org/10.1097/CM9.0000000000000589 | Wrong patient population  (healthy subjects) |
| 20 | Erratum: Protocol on transcranial alternating current stimulation for the treatment of major depressive disorder: A randomized controlled trial (Chinese Medical Journal (2020) 133:1 (61-67) DOI: 10.1097/CM9.0000000000000589). | Data copy |

**Supplementary Table 4b.** Excluded registered clinical trials in the second level screening.

|  | **Source Register** | **Reason for exclusion** |
| --- | --- | --- |
| 1 | [https://ClinicalTrials.gov/show/NCT01909011](https://clinicaltrials.gov/show/NCT01909011) | Ineligible topic |
| 2 | https://ClinicalTrials.gov/show/NCT04465136 | Ineligible topic |
| 3 | http://en.irct.ir/trial/48694 | Wrong patient population |
| 4 | https://clinicaltrials.gov/show/NCT03800030 | Wrong patient population |
| 5 | https://clinicaltrials.gov/show/NCT04560959 | Wrong patient population |
| 6 | https://clinicaltrials.gov/show/NCT04231825 | Wrong patient population |
| 7 | https://clinicaltrials.gov/show/NCT05664412 | Wrong patient population |
| 8 | https://clinicaltrials.gov/show/NCT04036630 | Wrong patient population |
| 9 | https://clinicaltrials.gov/show/NCT05446350 | Wrong patient population |
| 10 | https://clinicaltrials.gov/show/NCT04680481 | Wrong patient population |
| 11 | https://ClinicalTrials.gov/show/NCT03305328 | Wrong patient population |
| 12 | http://en.irct.ir/trial/27922 | Wrong patient population |
| 13 | http://en.irct.ir/trial/52038 | Wrong patient population |
| 14 | https://anzctr.org.au/ACTRN12618001705279.aspx | Wrong patient population |
| 15 | http://itmctr.ccebtcm.org.cn/en-US/Home/ProjectView?pid=fa27b73e-0cef-4206-afd7-6837490f2f55 | Data copy |
| 16 | https://ClinicalTrials.gov/show/NCT02723188 | Wrong patient population |
| 17 | https://ClinicalTrials.gov/show/NCT03036319 | Wrong patient population |
| 18 | https://ClinicalTrials.gov/show/NCT04115215 | Wrong patient population |
| 19 | https://clinicaltrials.gov/show/NCT05399381 | Wrong patient population |
| 20 | http://www.ctri.nic.in/Clinicaltrials/pmaindet2.php?trialid=33445 | Ineligible topic |
| 21 | https://ClinicalTrials.gov/show/NCT03475446 | Wrong patient population |
| 22 | http://www.drks.de/DRKS00024035 | Wrong outcomes |
| 23 | http://www.drks.de/DRKS00008209 | Wrong patient population |
| 24 | https://ClinicalTrials.gov/show/NCT05159336 | Wrong patient population |
| 25 | http://www.chictr.org.cn/showproj.aspx?proj=32964 | Wrong patient population |
| 26 | https://anzctr.org.au/ACTRN12619000870156.aspx | Duplicate |
| 27 | https://ClinicalTrials.gov/show/NCT05251649 | Duplicate |
| 28 | https://ClinicalTrials.gov/show/NCT03412604 | Duplicate |
| 29 | https://ClinicalTrials.gov/show/NCT04870710 | Duplicate |
| 30 | [https://ClinicalTrials.gov/show/NCT04515433](https://clinicaltrials.gov/show/NCT04515433) | Duplicate |
| 31 | [https://ClinicalTrials.gov/show/NCT04425148](https://clinicaltrials.gov/show/NCT04425148) | Duplicate |
| 32 | https://ClinicalTrials.gov/show/NCT05203523 | Duplicate |
| 33 | https://ClinicalTrials.gov/show/NCT04842955 | Duplicate |
| 34 | https://ClinicalTrials.gov/show/NCT03122587 | Duplicate |
| 35 | http://www.drks.de/DRKS00020828 | Duplicate |
| 36 | https://ClinicalTrials.gov/show/NCT04277351 | Duplicate |
| 37 | https://ClinicalTrials.gov/show/NCT03449979 | Duplicate |
| 38 | https://ClinicalTrials.gov/show/NCT05198726 | Duplicate |
| 39 | [https://ClinicalTrials.gov/show/NCT02360228](https://clinicaltrials.gov/show/NCT02360228) | Duplicate |
| 40 | https://ClinicalTrials.gov/show/NCT03290326 | Duplicate |
| 41 | https://ClinicalTrials.gov/show/NCT05569902 | Duplicate |
| 42 | https://ClinicalTrials.gov/show/NCT03221270 | Duplicate |
| 43 | http://www.chictr.org.cn/showproj.aspx?proj=22048 | Duplicate |
| 44 | [https://ClinicalTrials.gov/show/NCT04545294](https://clinicaltrials.gov/show/NCT04545294) | Duplicate |
| 45 | https://ClinicalTrials.gov/show/NCT03221413 | Duplicate |
| 46 | [https://ClinicalTrials.gov/show/NCT02339285](https://clinicaltrials.gov/show/NCT02339285) | Duplicate |
| 47 | https://ClinicalTrials.gov/show/NCT03920826 | Duplicate |

**Supplementary Table 5a.** Joanna Briggs Institute (JBI) checklist for randomized control trials (RCTs).

| **RCTs** | | | | | | | | | | | | | |
| --- | --- | --- | --- | --- | --- | --- | --- | --- | --- | --- | --- | --- | --- |
| **Study** | **Q1** | **Q2** | **Q3** | **Q4** | **Q5** | **Q6** | **Q7** | **Q8** | **Q9** | **Q10** | **Q11** | **Q12** | **Q13** |
| Ahn, 2019 | Yes | Yes | Yes | Yes | Yes | Yes | Yes | Yes | Yes | Yes | Yes | Yes | Yes |
| Alexander, 2019 | Yes | Yes | Yes | Yes | Yes | Yes | Yes | Yes | Yes | Yes | Yes | Yes | Yes |
| Assogna, 2021 | Yes | Yes | N/A | Yes | Yes | Yes | Yes | Yes | Yes | N/A | N/A | Yes | Yes |
| Benussi, 2021 | Yes | Yes | Yes | Yes | Yes | Yes | Yes | Yes | Yes | Unclear | Yes | Yes | Yes |
| Benussi, 2022 | Yes | Yes | Yes | Yes | Yes | Yes | Yes | Yes | Yes | unclear | Yes | Yes | Yes |
| Chang, 2021 | Yes | Yes | Yes | Yes | Yes | Yes | Yes | Yes | Yes | Yes | Yes | Yes | Yes |
| Daughters, 2022 | Yes | Yes | Unclear | Yes | Yes | Yes | Yes | Yes | Yes | Yes | Yes | Yes | Yes |
| Davis, 2023 | Yes | Yes | Yes | Yes | Yes | Yes | Yes | Yes | Yes | Yes | Yes | Yes | Yes |
| Davis, 2023 | Yes | No | Yes | Unclear | No | Yes | Yes | Yes | Yes | Unclear | Yes | Yes | Yes |
| Del Felice, 2019 | Yes | Yes | Yes | Yes | No | Yes | No | Yes | Yes | Yes | Yes | Yes | Yes |
| Kannen K, 2022 | Yes | Yes | Unclear | Yes | No | Yes | No | Yes | Yes | Unclear | Yes | Yes | Yes |
| Kehler, 2020 | N/A | N/A | N/A | N/A | N/A | N/A | N/A | N/A | N/A | N/A | N/A | Yes | Unclear |
| Kim J, 2021 | Yes | Yes | Yes | Yes | Yes | Yes | Yes | Yes | Yes | Yes | Yes | Yes | Yes |
| Leite J et al. (2022) | Yes | Yes | N/A | Yes | Yes | Yes | Yes | Yes | Yes | N/A | N/A | Yes | Yes |
| McAleer , 2023 | Yes | Yes | Yes | Yes | Unclear | Yes | Yes | Yes | Yes | Unclear | Yes | Yes | Yes |
| Mellin, 2018 | Yes | Yes | Yes | Yes | Yes | Yes | Yes | Yes | Yes | Yes | Yes | Yes | Yes |
| Mondino, 2020 | Yes | Yes | Unclear | Yes | Yes | Yes | Yes | Yes | Yes | Yes | Yes | Yes | Yes |
| Motamedi, 2023 | Yes | Yes | Unclear | Yes | Yes | Yes | Yes | Yes | Yes | Yes | Yes | Yes | Yes |
| Naro, 2016 | Yes | Yes | No | Yes | Unclear | Yes | Yes | Yes | Yes | Yes | Yes | Yes | Yes |
| Palm, 2022 | Yes | Yes | Yes | Yes | Yes | Yes | No | Yes | Yes | Yes | Yes | Yes | Yes |
| Riddle, 2022 | Yes | Yes | Unclear | Yes | Yes | Yes | Yes | Yes | Yes | Yes | Yes | Yes | Yes |
| Rufener, 2019 | Yes | Unclear | Yes | Unclear | Unclear | Yes | Unclear | Yes | Yes | Yes | Yes | Yes | Yes |
| Wang, 2022 | Yes | Yes | Yes | Yes | Yes | Yes | Yes | Yes | Yes | Yes | Yes | Yes | Yes |
| Wang, 2020 | Yes | Yes | Yes | Yes | Yes | Yes | Yes | Yes | Yes | Yes | Yes | Yes | Yes |
| Xing, 2020 | Yes | Yes | N/A | Yes | Yes | Yes | Yes | Yes | Yes | N/A | N/A | Yes | Yes |
| Yeh, 2023 | Yes | Yes | Yes | Yes | Yes | Yes | Yes | Yes | Yes | Yes | Yes | Yes | Yes |
| Zhang, 2022 | Yes | Yes | Yes | Yes | Yes | Yes | Yes | Yes | Yes | Yes | Yes | Yes | Yes |
| Zhou Q, 2021 | Yes | Yes | Unclear | Yes | Yes | Yes | Yes | Yes | Yes | Yes | Yes | Unclear | Yes |
| Zhou, 2022 | Yes | Yes | Yes | Yes | Yes | Yes | Yes | Yes | Yes | Yes | Yes | Yes | Yes |
| Dallmer-Zerbe, 2020 | Yes | Yes | Unclear | Unclear | No | Yes | Unclear | Yes | Yes | Unclear | Yes | Yes | Yes |
| Hoy, 2016 | Yes | Yes | Yes | Yes | No | Yes | No | Yes | Yes | Yes | Yes | Yes | Yes |
| Huang, 2021 | Yes | Yes | N/A | Yes | Yes | Yes | Yes | Yes | Yes | N/A | N/A | Yes | Yes |
| Jacobson, 2022 | Yes | Yes | N/A | Yes | Yes | Yes | Yes | Yes | Yes | N/A | N/A | Yes | Yes |
| Kehler, 2020 | Yes | Unclear | Unclear | Unclear | Unclear | Yes | Yes | Yes | Yes | Yes | Yes | Yes | Unclear |
| Leite, 2022 | Yes | Yes | N/A | Yes | Yes | Yes | Yes | Yes | Yes | N/A | N/A | Unclear | Yes |
| Marchesotti, 2020 | Yes | Yes | Unclear | Yes | No | Yes | No | Yes | Yes | Unclear | Yes | Yes | Yes |

Q1: Was true randomization used for the assignment of participants to treatment groups? Q2: Was allocation to treatment groups concealed? Q3: Were treatment groups similar at the baseline? Q4: Were participants blind to treatment assignment? Q5: Were those delivering treatment blind to treatment assignment? Q6: Were treatment groups treated identically other than the intervention of interest? Q7: Were outcomes assessors blind to treatment assignment? Q8: Were outcomes measured in the same way for treatment groups? Q9: Were outcomes measured in a reliable way Q10: follow-up complete, and if not, were differences between groups in terms of their follow-up adequately described and analyzed? Q11: Were participants analyzed in the groups to which they were randomized? Q12: Was appropriate statistical analysis used? Q13: Was the trial design appropriate, and any deviations from the standard RCT design (individual randomization, parallel groups) accounted for in the conduct and analysis of the trial?

**Supplementary Table 5b.** JBI checklist for quasi-experimental studies.

| **Quasi-Experimental Studies** | | | | | | | | | |
| --- | --- | --- | --- | --- | --- | --- | --- | --- | --- |
| **Study** | **Q1** | **Q2** | **Q3** | **Q4** | **Q5** | **Q6** | **Q7** | **Q8** | **Q9** |
| Sprugnoli, 2021 | Yes | N/A | N/A | No | Yes | Yes | Yes | Yes | Yes |
| Werchowski, 2022 | Yes | Unclear | Yes | Yes | Yes | Yes | Yes | Yes | Yes |
| Moussavi, 2021 | Yes | Yes | Yes | Yes | Yes | Unclear | Yes | Yes | Unclear |
| Raymond, 2023 | Yes | Yes | Yes | No | Yes | Yes | Yes | Yes | Yes |

Q1: Is it clear in the study what is the ‘cause’ and what is the ‘effect’ (i.e. there is no confusion about which variable comes first)? Q2: Were the participants included in any comparisons similar? Q3: Were the participants included in any comparisons receiving similar treatment/care, other than the exposure or intervention of interest? Q4: Was there a control group? Q5: Were there multiple measurements of the outcome, both pre and post intervention/exposure? Q6: Was follow up complete and if not, were differences between groups in terms of their follow up adequately described and analyzed? Q7: Were the outcomes of participants included in any comparisons measured in the same way? Q8: Were outcomes measured in a reliable way? Q9: Was appropriate statistical analysis used?

**Supplementary Table 5c.** JBI checklist for case series.

| **Case Series** | | | | | | | | | | |
| --- | --- | --- | --- | --- | --- | --- | --- | --- | --- | --- |
| **Study** | **Q1** | **Q2** | **Q3** | **Q4** | **Q5** | **Q6** | **Q7** | **Q8** | **Q9** | **Q10** |
| Dhaynaut, 2022 | Yes | Yes | Yes | Unclear | Unclear | Yes | Yes | Yes | No | Yes |
| Haller, 2020- 1 | Unclear | Unclear | Yes | No | No | No | Yes | Yes | No | Yes |
| Haller, 2020-2 | No | Yes | Yes | Unclear | Unclear | No | Yes | Yes | No | Yes |
| Haller, 2020-3 | No | Yes | Unclear | Unclear | Unclear | No | No | Yes | No | Yes |
| Kallel 2016 | No | Yes | Yes | No | No | No | No | Yes | No | Yes |
| Riddle, 2022 | No | Unclear | Unclear | No | No | No | No | Yes | No | Yes |
| Soleimani, 2022 | Yes | Unclear | Unclear | Yes | Yes | Yes | Unclear | Yes | Yes | Yes |
| Sreeraj, 2020 | Yes | Yes | Yes | No | No | Unclear | Yes | Yes | Unclear | Yes |

Q1: Were there clear criteria for inclusion in the case series? Q2: Was the condition measured in a standard, reliable way for all participants included in the case series? Q3: Were valid methods used for the identification of the condition for all participants included in the case series? Q4: Did the case series have consecutive inclusion of participants? Q5: Did the case series have complete inclusion of participants? Q6: Was there clear reporting of the demographics of the participants in the study? Q7: Was there clear reporting of clinical information of the participants? Q8: Were the outcomes or follow-up results of cases clearly reported? Q9: Was there clear reporting of the presenting site(s)/clinic(s) demographic information? Q10: ​​Was statistical analysis appropriate?

**Supplementary Table 5d.** JBI checklist for case reports.

| **Case Reports** | | | | | | | | |
| --- | --- | --- | --- | --- | --- | --- | --- | --- |
| **Study** | **Q1** | **Q2** | **Q3** | **Q4** | **Q5** | **Q6** | **Q7** | **Q8** |
| Force, 2021 | Unclear | No | Yes | No | Yes | Yes | Yes | Yes |
| Liu, 2022 | Yes | Yes | Yes | Yes | Yes | Yes | Yes | Yes |
| Riddle J, 2020 | No | Yes | Yes | Yes | Yes | Yes | No | Yes |
| Sreeraj, 2019 | Unclear | Unclear | Yes | Yes | Yes | Yes | Yes | Yes |
| Sreeraj, 2017 | Unclear | Unclear | Yes | Yes | Yes | Yes | Yes | Yes |
| Wilkening, 2019 | No | Yes | Yes | Yes | Yes | Yes | Yes | Yes |
| Brechet, 2021 | No | Yes | Yes | Yes | Yes | Yes | Yes | Yes |

Q1:Were patients’ demographic characteristics clearly described? Q2: Was the patient’s history clearly described and presented as a timeline? Q3: Was the current clinical condition of the patient on presentation clearly described? Q4: Were diagnostic tests or assessment methods and the results clearly described? Q5: Was the intervention(s) or treatment procedure(s) clearly described? Q6: Was the post-intervention clinical condition clearly described? Q7: Were adverse events (harms) or unanticipated events identified and described? Q8: Does the case report provide takeaway lessons?

| **a.** | **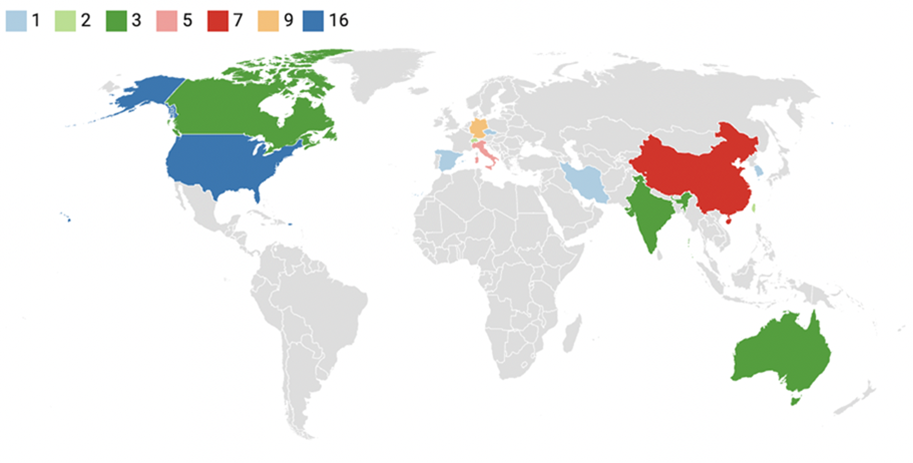** |
| --- | --- |
| **b.** | **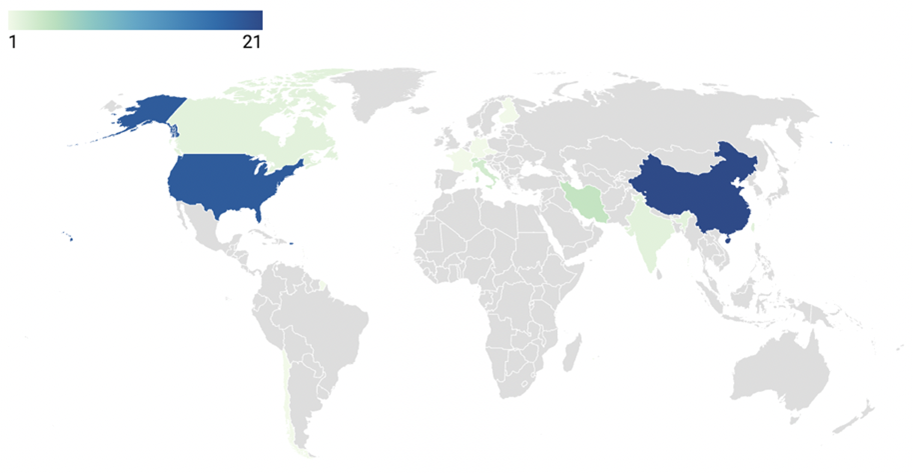** |

Supplementary Figure 1. (a) Distribution of published studies by country. Following the United States and Germany, tACS studies were also conducted in China (13.2%), Italy (9.4%), Australia (5.7%), Canada (5.7%), India (5.7%), Taiwan (3.8%), Korea (1.9%), Spain (1.9%) and Switzerland (1.9%). (b) Distribution of registered tACS clinical trials by country. After the United States, the percentage of registered trials in Australia was 8.57%, while Germany and Iran each contributed to 5.71% of trials. Italy and Switzerland contributed 4.29% each, with Canada and India accounting for 2.86% of trials each. Taiwan contributed to 2.86% of trials, and Czechia, Finland, France, and Chile each made smaller contributions to 1.43% of trials.
